# Gold(III) Complexes with 2-(1-Ethylbenzyl)pyridine as Promising Antimicrobial and Antitumor Agents

**DOI:** 10.3390/molecules30071611

**Published:** 2025-04-04

**Authors:** Antonio Zucca, Bruna Canu, Maria I. Pilo, Sergio Stoccoro, Giacomo Senzacqua, Sara Fais, Giuseppina Pichiri, Alessandra Scano

**Affiliations:** 1 Department of Chemical, Physical, Mathematical and Natural Sciences, University of Sassari, Via Vienna 2, 07100 Sassari, Italy; brunacanu@hotmail.it (B.C.); mpilo@uniss.it (M.I.P.); stoccoro@uniss.it (S.S.); g.senzacqua@studenti.uniss.it (G.S.); 2 Consorzio Interuniversitario Reattività Chimica e Catalisi (CIRCC), 70126 Bari, Italy; 3 Department of Surgical Sciences, University of Cagliari, 09124 Cagliari, Italy; sara.fais@unica.it; 4 Department of Medical Sciences and Public Health, University of Cagliari, 09124 Cagliari, Italy; pichiri@unica.it

**Keywords:** Gold(III) cyclometalated compounds, nitrogen ligands, antimicrobial complexes, laboratory medicine

## Abstract

Antimicrobial resistance (AMR) is one of the most urgent public health problems worldwide; multidrug resistance (MDR) is also of concern. In an effort to find new classes of antibiotics, recent studies have found that coordination compounds of noble metals show promising biological effects both in vitro and in vivo, deserving attention as a new class of possible antimicrobial agents. Metal ions in biological systems can essentially have two roles: structural or functional. In the former, the metal ion serves to stabilize structures, especially proteins, while in the latter, the metal is involved in bio-site reactivity (essentially in metallo-enzymes). Two new complexes with 2-(1-ethyl-benzyl)pyridine (py^eb^), one monodentate adduct and one cyclometalated ([Au(py^eb^)Cl_3_] and [Au(py^eb^-H)Cl_2_], respectively), have been synthesized, characterized, and tested against Gram-positive and Gram-negative bacteria, as well as yeasts, revealing promising antibacterial and antibiofilm properties. The two complexes have been thoroughly characterized by means of 1D and 2D NMR spectroscopy, as well as by cyclic voltammetry, conductivity measurements, FT-IR, and elemental analysis. The study showed that the two derivatives are structurally and chemically different, with the cyclometalated complex being chemically and electrochemically more stable. Antimicrobial assays demonstrated that solutions of the monodentate adduct and of the cyclometalated complex have inhibitory and antibiofilm effects against the pathogenic bacteria *E*. *coli*, *K*. *pneumoniae*, *S*. *aureus*, and *S*. *pyogenes* but were unable to reveal a fungicidal effect on *C*. *albicans*. A preliminary study was conducted to assess the anti-cancer activity of the compounds, and treatments with the gold compounds also resulted in a significant reduction in the metabolic activity of HT29 colon cancer cells.

## 1. Introduction

Pyridine and its derivatives occupy a privileged place among heterocycles; these derivatives, which are an integral part of various natural products, may be easily modified in different ways on the heterocyclic ring to provide a multitude of molecules with a variety of properties in several fields. In particular, these compounds have emerged as promising candidates in the field of biomedical research, exhibiting a wide array of applications, e.g., as antitumor, antimicrobial, antiviral, anti-inflammatory, analgesic, anticonvulsant, anti-Alzheimer’s, anti-ulcer, or antidiabetic agents [1,2,3].

In addition to their properties as free organic compounds, pyridine derivatives constitute one of the most important classes of ligands in coordination chemistry, generating complexes widely studied in an array of fields, spanning from catalysis and material science to biomedicine [4].

Particularly attractive appear the organometallic complexes formed by cyclometallation. These derivatives display an intrinsic stability related to the chelate effect and possess an easy tunability of properties. Several families of cyclometalated pyridine-derived complexes are known, such as classical N^C [5], terdentate N^N^C [6] or N^C^N [7] pincer complexes, or the uncommon “rollover complexes” [8].

Among the various fields of application of pyridine complexes, their antimicrobial activity is one of the most recent and appears to be one of the most promising. This is related to antimicrobial resistance (AMR), which has become one of the most pressing public health problems worldwide and is on the way to becoming the leading cause of death in the coming decades [9]. The escalation of antimicrobial resistance requires new strategies to combat disease-causing bacteria and yeasts. For this reason, new approaches to develop the next generation of antimicrobials have become imperative.

Metal-based compounds have emerged as a potential alternative to traditional organic antimicrobial drugs, generating the new class of metalloantibiotics [10]. In particular, cyclometalated gold(III) complexes have shown potential applications as antimicrobial drugs [11]. Reports on the antimicrobial activity of gold complexes are much fewer, even though metal-based compounds represent a very promising chemical scaffold in this field, since they can act against nonclassical targets and multiple bacterial sites simultaneously.

**Chart 1:**
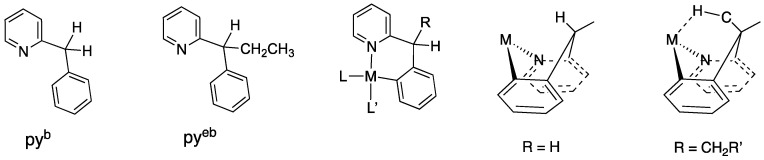
Six-membered cyclometalated ring with boat-like conformation, in the absence (R = H) and presence of a substituent (e.g., R = CH_3_) on the benzylic carbon.

An especially interesting family of ligands in this respect is constituted by 2-benzylpyridines and 6-benzyl-2,2′-bipyridines, which, after deprotonation, can behave as six-membered cyclometalated ligand, with N^C and N^N^C sequences of donor atoms [12,13]. The cyclometalated complexes of benzyl pyridines, as well as those of benzyl 2,2′-bipyridines, have an important feature: the substituents on the benzyl carbon play a significant role in the chemical behavior of the complex, although being apparently far from the metal center. This is due to the fact that the six-membered C^N cycle assumes a boat conformation, and substituents on the benzyl carbon, such as a methyl, can interact with the metal center through an interaction still not well understood, variously interpreted as pre-agostic, anagostic, etc. (Chart 1) [14,15]. Several X-ray studies have shown the presence of metal-hydrogen short distances, where a hydrogen on the benzyl substituent is located close to the metal (Pt, Pd or Au) in an apical position, with metal-hydrogen distances below the sum of their van der Waals radii [16].

**Chart 2:**
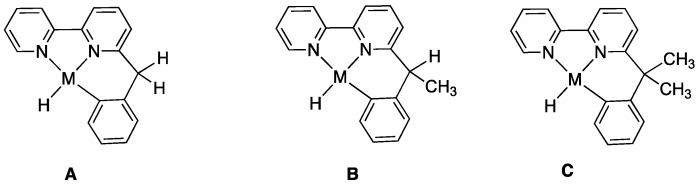
Comparison between three Pt(II) hydrides. Complex **A**, extremely unstable, highly reactive, complex **C**, highly stable and less reactive, complex **B** in the middle.

As an example, in platinum(II) hydride complexes derived from 6-benzy-2,2′-bipyridines [17], a considerable difference in reactivity and stability was observed between the three complexes A, B, and C shown in Chart 2. The difference is also reflected by markedly different Pt-H coupling constants in the ^1^H NMR spectra, reflecting very different Pt-hydride bonds in the three complexes. For this reason, the substitution of a hydrogen on the benzyl carbon with a substituent capable of providing metal interactions is not a trivial substitution.

The simplest ligand in this family, 2-benzylpyridine (py^b^, Chart 1), has been studied in the past, giving, among the others, the gold(III) cyclometalated complex [Au(py^b^-H)Cl_2_] [18]. Successively, the derived gold(III) cyclometalated complex, [Au(py^b^-H)(mnt)] (mnt = 1,2-dicyanoethene-1,2-dithiolate), was tested against a panel of Gram-positive and Gram-negative bacteria, as well as three yeasts, showing a remarkable bacteriostatic antimicrobial activity against staphylococci, with minimum inhibitory concentration (MIC) values of 1.56 and 3.13 μg/mL for *S*. *haemoliticus* and *S*. *aureus*, respectively [19]. Recently, Casini and coworkers took up the study of this ligand in a thorough paper in which they show that the cyclometalated complex [Au(py^b^-H)Cl_2_] is active on a series of Gram-positive bacterial strains, including *B*. *subtilis* and *S*. *aureus;* the [Au(py^b^-H)Cl_2_] complex showed a complex mode of antibacterial action, not including induction of oxidative stress or cell membrane damage, activating cellular stress response routes different from those of other metal-based complexes. Finally, the complex appeared to be poorly toxic to mouse liver and kidney tissues, making it a worthy antibacterial drug candidate [20].

Addition to a substituent on the benzylic carbon of 2-benzylpyridine furnishes the possibility of adding an axial M---H-C pre-agostic interaction to the cyclometalated complex, modifying the chemical and biological behavior. For this reason, our interest has been directed toward a new ligand, 2-(1-ethylbenzyl)pyridine (py^eb^, Chart 1), whose ethyl substituent on the benzylic carbon may furnish opportunities for C-H bond interactions and affect the properties of the obtained complexes, in particular, as antimicrobials.

## 2. Results and Discussion

The 2-(1-ethylbenzyl)pyridine ligand was synthesized starting from 2-phenylbutyronitrile, through an acetylene co-cyclotrimerization reaction in the presence of cobalt Bonneman’s catalyst, a useful method for the build-up of pyridine rings (see experimental). The ligand has been previously prepared following other synthetic routes [21,22]. However, a full NMR characterization has never been made, so we analyzed in depth 2-(1-ethylbenzyl)pyridine by means of ^1^H and ^13^C mono- and two-dimensional NMR, which allowed a complete assignment of proton and carbon resonances.

**Scheme 1:**
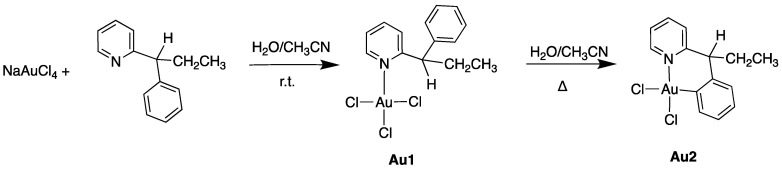
Synthesis of complexes **Au1** and **Au2**.

Reaction of 2-(1-ethylbenzyl)pyridine with Na[AuCl_4_] in a H_2_O/acetonitrile mixture gave at room temperature a bright yellow precipitate, isolated and characterized as the adduct [Au(py^eb^)Cl_3_], **Au1**, in fairly good yield (Scheme 1).

The ^1^H NMR spectrum of **Au1** in CDCl_3_ was in agreement with the given formulation, showing only one set of signals. The H^6^ proton is usually taken as a reference for coordination, being adjacent to the pyridine nitrogen. However, in this case, the signal shifted only to 8.69 ppm after coordination (Δδ = 0.1 ppm), a value which is in line with those previously reported by some of us for similar adducts [13]. At variance, several other signals are significantly deshielded after coordination, in particular, the H^4^ signal, which resonates at 8.04 ppm (Δδ = 0.48 ppm). The most deshielded signal, however, is the benzylic CH, which appears as a double doublet at 5.02 ppm with a considerable coordination shift of +1.03 ppm. This abnormal shift likely suggests a proximity of this hydrogen to the metal or to one of the coordinated chlorides. The CH_2_ group appears, as expected, as two diastereotopic protons, showing multiplets, only slightly shifted, at 2.38 and 2.18 ppm. A 2D COSY spectrum helped in the assignment of all ^1^H NMR signals, whereas a NOESY spectrum showed interesting features: the benzylic CH proton shows contacts with the H*ortho* on the phenyl ring, and not with the adjacent H^3^ on the pyridine; in addition, the two diastereotopic protons of the CH_2_ group are located in different environments, showing for one, at 2.15 ppm, in proximity with the phenyl H*ortho* protons, and for the other one, at 2.37 ppm, in proximity with the pyridine H^3^. These data suggest a hindered rotation of the benzylic substituent with different positions of the diastereotopic CH_2_ protons. The infrared spectrum of **Au1** shows a band at 366 cm^−1^, in the region expected for pyridine LAuCl_3_ adducts (Au-Cl stretching) [23].

When the reaction was conducted at reflux for 48 h, a whitish precipitate was formed and isolated in the solid state, characterized as the six-membered cyclometalated complex [Au(py^eb^-H)Cl_2_], **Au2**.

**Figure 1:**
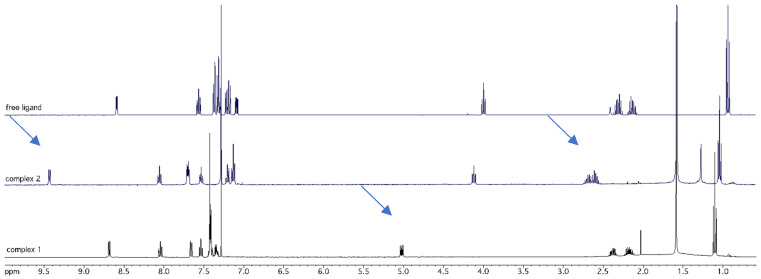
^1^H NMR spectra (CDCl_3_) of complexes **Au1** (**below**), **Au2** (**middle**), and free ligand (**above**). The arrows indicate the most significant shifts in the spectra.

The ^1^H NMR spectrum of **Au2** (Figure 1) shows the presence of only eight hydrogens in the aromatic zone, in place of the nine hydrogens of the free ligand and the adduct **Au1**. The proton H^6^ is significantly shifted, resonating at 9.43 ppm (Δδ = 0.84 ppm). The benzyl hydrogen is found at 4.12 ppm, while the two hydrogens of the CH_2_ group appear shifted to lower fields (δ = 2.67 and 2.60, Δδ = 0.37, and 0.46 ppm, respectively).

**Figure 2:**
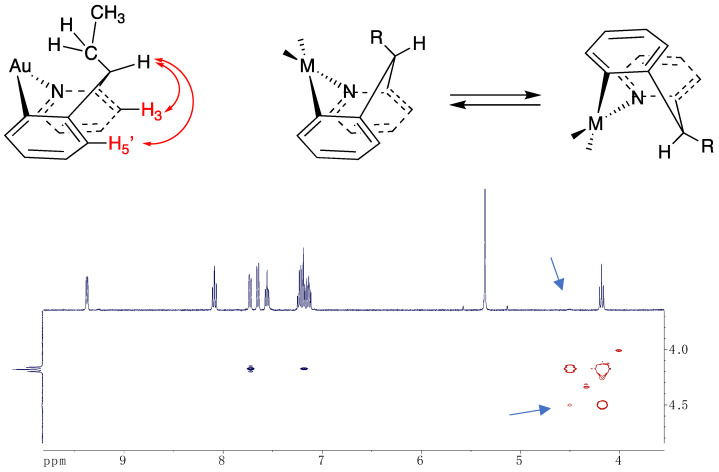
Section of the 2D NOESY/EXSY spectrum (positive values in blue, negative in red), showing in blue (positive values) NOE contacts between the benzylic C-H (4.18 ppm) and H^3^ and H^5′^, in red (negative values) exchange of the same benzylic C-H between the main conformer (ca 98%) and the minor (ca 2%), barely visible in the ^1^H NMR spectrum (indicated by the arrows).

In the absence of an X-ray crystal structure, complex **Au2** was thoroughly studied by means of 1-D and 2-D NMR spectroscopy. ^1^H 2D-COSY and NOESY experiments in CD_2_Cl_2_ solution allowed a complete assignment of ^1^H data. In particular, the NOESY spectrum shows NOE contacts between the benzylic C-H and the adjacent H^3^ and H^5′^ protons (Figure 2). No contacts are visible between the CH_2_ and CH_3_ protons and the aromatic region. This indicates that the six-membered cyclometalated ring adopts a semi-rigid boat conformation with the ethyl group pointing toward the metal, in axial position, with possible Au---H interactions, supported by the deshielding of the CH_2_ protons, ca. 0.4–0.5 ppm. In addition, the EXSY section of the NOESY-EXSY spectrum (cross peaks with negative values) demonstrates a flipping interconversion between the main species (ca. 98%) and a secondary species (ca. 2%), which is attributable to a conformer of complex **Au2** having the C-H in axial position and the ethyl group equatorial (Figure 2).

The presence of two conformers was observed for the analogous Au(N^C)Cl_2_ complex derived from 2-1-methlbenzyl)pyridine [13], where a 4:1 molar ratio between the two conformers was observed, reflecting an extremely different relative stability between the conformers in solution and confirming the influence of the substituent of the benzylic carbon. The ^13^C NMR spectrum shows all the expected signals, and ^1^H-^13^C 2D HSQC and long-range HMBC spectra completed the characterization and assignment of all ^13^C NMR data (see experimental) allowing, inter alia, the identification of the metalated carbon, at 139.88 ppm.

The electrochemical behavior of complexes **Au1** and **Au2** has been investigated by cyclic voltammetry (CV) in methylene chloride and in acetonitrile, using tetraethylammonium hexafluorophosphate (TEAPF_6_) as the supporting electrolyte. In all cases, no responses have been observed in the direct anodic scan. The voltammetric experiments have been first performed in methylene chloride to avoid the occurrence of coordination by the solvent molecules. The cyclic voltammetry response of **Au1** (Figure S17, continuous curve), at 0.20 V s^−1^ as the potential scan rate, shows three cathodic processes located at −0.33 V, −0.64 V, and −0.83 V, respectively, with associated backward peaks at about 0.1 V, 0.5 V, and 0.7 V. Slight and reproducible differences are observed in the subsequent scans. In particular, the second peak (−0.64 V) is shifted at more cathodic values (about 60 mV), showing higher current intensity. Furthermore, a thin gold film is observed on the electrode surface at the end of the cathodic scan. The pattern of the whole voltammetric response suggests a two-step reduction process, Au(III)→Au(I), presumably involving a rearrangement of the ligand sphere, followed by a final Au(I)→Au(0) reduction. A chloride ligand detachment and subsequent oxidation are suggested by the broad anodic peak at about 0.7 V in the reverse scan, whereas no re-oxidation of Au(0) is observed before the solvent discharge.

Similarly, the voltammetric behavior of **Au1** in acetonitrile (Figure S17, dotted curve) as the solvent shows three cathodic processes at −0.14 V, −0.44 V, and −0.81 V, respectively. Again, the appearance of a thin gold film is observed at the end of the cathodic scan, with a consequent shift of the last peak (−0.81 V) at slightly less negative values in the subsequent voltammetric cycles. Accordingly, the more cathodic process can be reasonably ascribed to the monoelectronic reduction of the Au(I) species derived by the less cathodic processes. Moreover, the reverse scan shows a peak at 0.02 V, a further broad, low current peak at about 0.5 V, and, interestingly, a last sharp peak at 0.92 V, whose shape is typical of an oxidative desorption of Au(0) [24].

**Figure 3:**
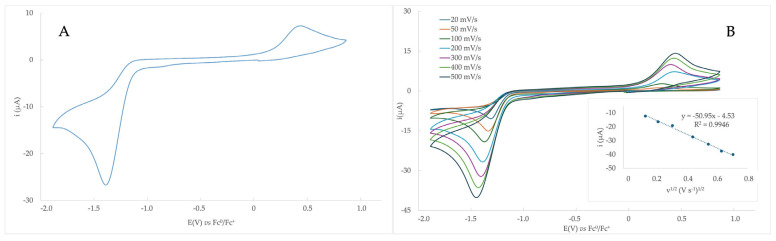
(**A**) Cyclic voltammetry response of 2 × 10^−3^ M **Au2** solution in CH_2_Cl_2_ containing 0.1 M TEAPF_6_ as supporting electrolyte; working electrode: Pt disk (diameter 2 mm); potential scan rate = 0.20 V s^−1^. (**B**) CV curves of **Au2** at potential scan rates from 0.02 V s^−1^ to 0.50 V s^−1^, and (inset) relationship between peak current and potential scan rate square root.

The voltammetric response of the cyclometalated complex **Au2** is significantly different from that of **Au1**. In particular, in methylene chloride, only one irreversible process at −1.39 V is observed, with an associated peak in the reverse anodic scan at 0.44 V, at a potential scan rate of 0.20 V s^−1^ (Figure 3A). CV curves obtained by varying the potential scan rates from 0.02 to 0.50 V s^−1^ evidence a shift of the cathodic peak toward more negative potential values (Figure 3B), as expected for a non-reversible electron-transfer process. Moreover, a linear relationship between the peak current (i_p_) and the scan rate square root (v^1/2^) (Figure 3B, inset) suggests a diffusion-controlled process. Neither further peaks at more cathodic potential values nor a gold film on the electrode surface are observed.

**Chart 3:**
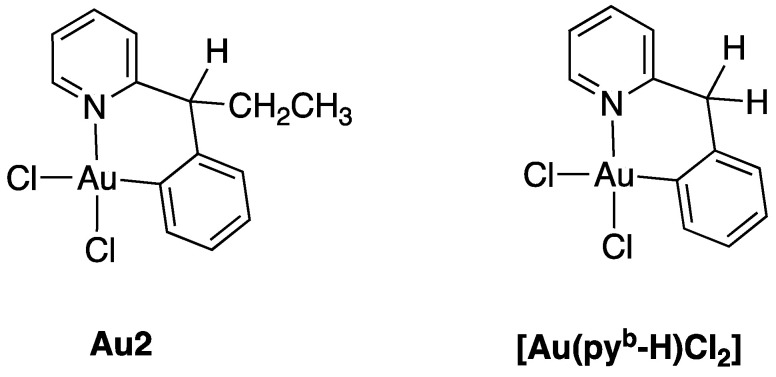
Comparison between **Au2**, [Au(py^eb^-H)Cl_2_], and the previously studied [Au(py^b^-H)Cl_2_].

**Figure 4:**
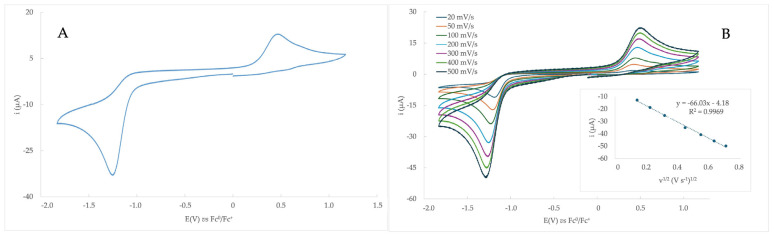
(**A**) Cyclic voltammetry response of 2 × 10^−3^ M **Au2** solution in CH_3_CN containing 0.1 M TEAPF_6_ as supporting electrolyte; working electrode: Pt disk (diameter 2 mm); potential scan rate = 0.20 V s^−1^. (**B**) CV curves of **Au2** at potential scan rates from 0.02 V s^−1^ to 0.50 V s^−1^, and (inset) relationship between peak current and potential scan rate square root.

The electrochemical behavior of **Au2** in acetonitrile (Figure 4A) is very similar to the case of methylene chloride used as the solvent, showing an irreversible cathodic process at −1.25 V and a backward peak at 0.47 V. The response still appears reproducible, suggesting a good stability of the complex also in acetonitrile in the voltammetric experiment timescale. The irreversible nature of the cathodic process, as well as its diffusive feature, are confirmed by the shift of the peak to more negative values at increasing scan rates (Figure 4B) and by the linear variation of i_p_ vs. v^1/2^ (Figure 4B, inset), respectively. Also in this case no deposition of metal gold is evidenced on the working electrode, despite the wider potential window available in acetonitrile than in methylene chloride. The comparison with the voltammetric behavior of **Au1** suggests formally ascribing the cathodic process of the cyclometalated complex to a bi-electronic reduction on the Au(III) center. E_p,c_-E_p/2,c_ values between 100 and 120 mV in acetonitrile have been observed, showing a slight dependence from the scan rate. This behavior is not completely in agreement with a totally irreversible electron-transfer process and suggests that a further chemical process can be coupled to the electrochemical one [25]. Furthermore, the range of E_p,c_-E_p/2,c_ values is wider in methylene chloride, maybe due to an involvement of the solvent in the associated chemical process. A similar behavior was observed for the analogue cyclometalated complex [Au(py^b^-H)Cl_2_], Ref. [18] (see Chart 3 for a comparison) at the same experimental conditions (acetonitrile as the solvent, 0.1 M TEAPF_6_ as the supporting electrolyte, 0.20 V s^−1^ as the potential scan rate). It is noteworthy that the E_p,c_ value of the py^eb.^ derivative **Au2** is 60 mV more negative than the py^b^ one (−1.25 V and −1.19 V, respectively, vs. Fc/Fc^+^). This cathodic shift suggests a higher stability of **Au2** than [Au(py^b^-H)Cl_2_] reasonably ascribable to the presence of the ethyl- substituent on the benzyl frame. Furthermore, the **Au2** cyclometalated complex is also considerably more stable than the adduct **Au1**, due to the chelating effect of the py^eb^ ligand. Finally, from a general point of view, both **Au1** and **Au2** are more stable in methylene chloride than in acetonitrile.

One important scope of this manuscript is the assessment of the antimicrobial properties of two structurally different gold(III) complexes, i.e., the monodentate adduct **Au1** and the cyclometalated complex **Au2**, respectively, and to analyze their different behaviors.

The conduct of these two complexes in solvents such as DMSO or water may be different, because, in general, monodentate adducts such as **Au1** have a less tightly bonded monodentate nitrogen ligand, which may be partially dislocated in the presence of good donors. In contrast, cyclometalated N^C ligands usually have a noteworthy stability due to the chelate effect and covalency of the gold-carbon bond. For this reason, we registered the ^1^H NMR spectra also in deuterated DMSO. The ^1^H spectrum of **Au2** is very neat, showing only one set of sharp signals, with chemical shift values related to the cyclometalated complex Au(N^C)Cl_2_. At variance, the ^1^H NMR spectrum of **Au1** in DMSO showed a dynamic behavior, in the NMR time scale: in addition to signals attributable to the adduct Au(py^eb^)Cl_3_, a second species, with large signals to a dynamic behavior in solution, is present. No free ligand is observed, so the dynamic behavior may be linked to chloride displacement by DMSO. Conductivity data, both in CH_2_Cl_2_ and DMSO initially showed no electrolyte behavior for complexes **Au1** and **Au2**. In the case of the adduct **Au1**, however, a slow increase in conductivity was observed in both solvents (c = 0.5 mM), with final values of 12.0 and 17.5 μS in CH_2_Cl_2_ and DMSO, respectively, suggesting a partial substitution of a chloride ligand. Solvolysis of chloride ligands is well known for cisplatin and cisplatin analogues in DMSO and water/DMSO solutions [26].

Even though complex **Au1** may have a dynamic behavior in DMSO, we deemed it of interest to investigate and compare the antimicrobial properties in solution of the two structurally different complexes **Au1** and **Au2**.

### 2.1. Antimicrobial Assay

The group of compounds represented by two gold(III) complexes: (i) the monodentate adduct **Au1** and (ii) the cyclometalated complex **Au2** solubilized in 1 mL dimethylsulfoxide (c = 0.01 M) were tested against Gram-positive (*Streptococcus pyogenes* DSM 20565, *Staphylococcus aureus* DSM 1104), Gram-negative (*Klebsiella pneumoniae* DSM 681, *Escherichia coli* DSM 1103), and yeast (*Candida albicans* DSM 1386) strains, revealing promising antibacterial and antibiofilm properties (DSM = German Collection of Microorganisms and Cell Cultures GmbH, Braunschweig, Germany).

### 2.2. Evaluation of Antibacterial Activity

Evaluation of the antimicrobial activity of the studied compounds was performed according to the procedures described by the Clinical and Laboratory Standards Institute (CLSI). A first line of evaluation was performed with the agar diffusion test (Kirby–Bauer). This procedure was useful for the rapid assessment of bacterial resistance or susceptibility to the different formulates. Briefly, for each bacterial strain, 20 mL of agarized agar medium (Microbiol, Uta, Cagliari, Italy) at 55 °C was added to a 90 mm Petri dish, and, before the agar solidified, four sterile iron rivets, 10 mm in diameter and 2 mm thick (Firm, Milan, Italy), were inserted into the agar mixture and then removed from the medium once it was cold. Under these conditions, each well contained 50 µL of solution of the tested compound. Each strain was inoculated onto the surface of the plate using a sterile buffer with a standardized bacterial inoculum of 5 × 10^7^ CFU for each bacterium and 5 × 10^6^ CFU for yeast. Three wells were used for each compound test and two for the negative control. Petri dishes were incubated in air at 37 °C for 24 h for aerobic strains and in 5% CO_2_ at 37 °C for microaerophilic species. After incubation, the diameter of the inhibition halo in mm was measured. The diameter of inhibition is proportional to the logarithm of the active concentration [mol/L] of the compound. Based on the Kirby–Bauer test, the compounds did not show any activity against *C*. *albicans* (inhibition diameter ~0 mm). However, activity of **Au1** and **Au2** was seen against the Gram-positive bacteria *S*. *aureus* and *S*. *pyogenes* and Gram-negative *E*. *coli* and *K*. *pneumoniae* (Table 1). In contrast, the free py^eb^ ligand did not show any antimicrobial activity under the same experimental conditions. Broth dilution and antibiofilm tests were performed only for compounds that showed activity. The results of the Kirby–Bauer diffusion procedure preliminary test indicated that the compounds showed similar antibacterial activity: they inhibited the same Gram-positive and Gram-negative strains and most importantly, they exerted a strong inhibiting action on the nosocomial infection-associated pathogen *K*. *pneumoniae* (Gram-negative). These preliminary results demonstrated that the compounds had an inhibitory effect against some pathogenic bacteria.

### 2.3. Minimum Inhibitory Concentration (MIC) and Minimum Bactericidal Concentration (MBC) Tests

The standard broth dilution method was used to study the antimicrobial efficacy of the compounds by evaluating the visible growth of microorganisms in nutrient broth. This evaluation was performed in sterile 96-well microplates, with each well containing serial dilutions (0.5% to 0.0004%) of each compound dissolved in nutrient broth. Briefly, serial concentrations of 0.01 M of each compound were tested, and the final concentration of the inoculum was 1 × 10^6^ CFU/mL for each tested strain. The experiment was repeated three times. After 24–48 h of incubation at 37 °C in an appropriate atmosphere (air or 5% CO_2_), the minimum inhibitory concentration (MIC) was defined as the lowest concentration of the tested compound that inhibited visible growth, i.e., the well showed the same absorbance (550 nm) of the negative control, measured with a Multiskan FC microplate photometer (Thermo Fisher Scientific IT, Milan, Italy). After determining the MIC of **Au1** and **Au2**, aliquots of 50 μL from all wells that showed no visible bacterial growth were seeded onto Mueller–Hinton agar plates (for *E*. *coli*, *K*. *pneumoniae*, and *S*. *aureus*) and Shaedler agar plates (for *S*. *pyogenes*) and incubated for 24 h at 37 °C. The minimum bactericidal concentration (MBC) is defined as the lowest concentration of an antimicrobial agent that kills 99.9% of the bacterial population.

### 2.4. Anti-Biofilm Assay

The minimum inhibitory concentration of biofilm (MBIC) was evaluated following the crystal violet staining protocol described by the Montana University Center for Biofilm Engineering (http://www.biofilm.montana.edu, accessed on 31 March 2025) with some modifications. Therefore, after 48 h of incubation in an appropriate atmosphere (air or 5% CO_2_), the medium was discarded, and the wells were gently washed three times with a 0.9% NaCl solution; then, 0.1 mL of a 0.1% crystal violet solution was added to each well; after 10 min, the dye was discarded, followed by three washes with 0.9% NaCl, and the dye linked to bacteria was then solubilized with 200 µL acetic acid (30%). Finally, the absorbance of the biofilm was measured at 620 nm with a Multiskan FC microplate photometer (Thermo Fisher Scientific IT, Milan, Italy). The MBIC represents the lowest concentration showing an absorbance comparable with the negative control (sample without bacteria). The antimicrobial-antibiofilm analysis, evaluated as previously described, showed appreciable activity (Table 2).

According to the Kirby–Bauer method, only the strains sensitive to **Au1** and **Au2** were used for MIC and MBC tests. As reported in Table 2, these compounds caused bacterial growth inhibition (MIC) in a concentration range from 2.5 × 10^−3^ M to 1.5 × 10^−4^ M. *S*. *aureus* and *K*. *pneumoniae* represented the most sensitive strains. In this work, Au1 and Au2 also showed bactericide activity. Table 2 also reports the minimum biofilm inhibition concentration between **Au1** and **Au2**. A significant difference between the two compounds in the ability to interfere in biofilm formation was observed in *S*. *aureus*. As shown in Table 2, **Au2** reported lower MBIC values (1.5 × 10^−4^) than **Au1** (2.5 × 10^−3^). In this context, the antibiofilm propriety could be interesting since these microbial species are frequently associated with “biofilm-related diseases” [27]; in fact, in both in vitro and in vivo experiments, their microbial sessile status is often more resistant to conventional biocides than in their planktonic form [28].

### 2.5. Preliminary Evaluation of Potential Cytotoxicity and Antitumor Activity

Gold(III) compounds with nitrogen ligands have found increasing interest for their antitumor properties [29,30,31,32,33,34]. For this reason, we also started a preliminary investigation on the cytotoxic and antitumor behavior of compounds **Au1** and **Au2**.

We used standard protocols, based on tissue cells in vitro, to observe the cell growth, reproduction, and morphological effects due to these compounds. In this context, the in vitro procedure that was used was the MTT assay (MTT = 3-(4,5-dimethyl-2-thiazolyl)- 2,5-diphenyl-2H-tetrazolium bromide methyl thiazolyl tetrazolium), which is a colorimetric assay for rapid evaluation of cell proliferation and cytotoxicity to gauge cell metabolism or function. The tetrazole ring can be broken by mitochondrial dehydrogenase in the cytochrome b and c sites of living cells, which reduces the yellow, water-soluble MTT to create a purple crystalline formazan. This compound is water-insoluble but soluble in dimethyl sulfoxide and other organic solvents. Measurement of the absorbance (optical density) colorimetric value reflects the number of surviving cells and metabolic activity, and the amount of crystals created has a positive association with the number of cells and their activity. This protocol describes a quick and reliable screening method that is suitable for the examination of the impact of other compounds on cell survival and their growth inhibition. For this reason, we tested **Au1** and **Au2** complexes in vitro using the HT29 colon cancer cell line. Commercial human cell line HT29 (ATCC, https://www.atcc.org, accessed on 31 March 2025), was obtained from the Istituto Nazionale per la Ricerca sul Cancro c/o CBA (ICLC, Genova, Italy). Confluent HT29 cells were isolated using trypsin/EDTA, and 2–3 × 10^4^ cells/cm^2^ were plated with a mixture of MEM (EBSS), 10% fetal bovine serum (FBS), 100 units/mL penicillin, 100 µg/mL streptomycin, and 2 mM L-Glutamine ND 1% non-essential amino acids.

**Figure 5:**
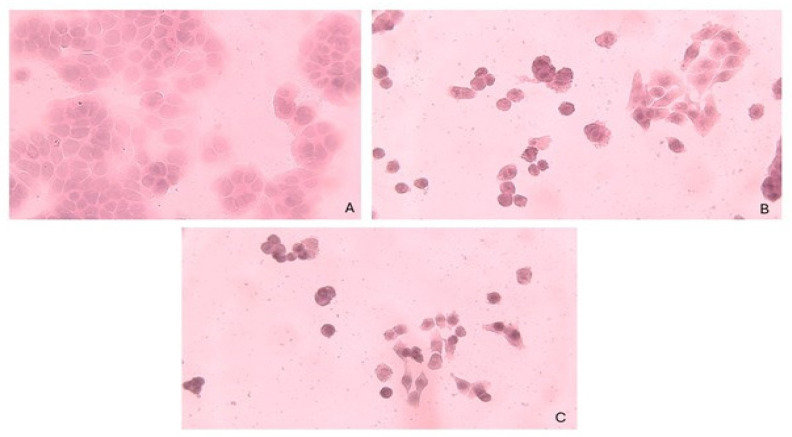
Effect on colon cancer cell line HT29 (**A**) of complexes **Au1** (**B**) and **Au2** (**C**).

**Figure 6:**
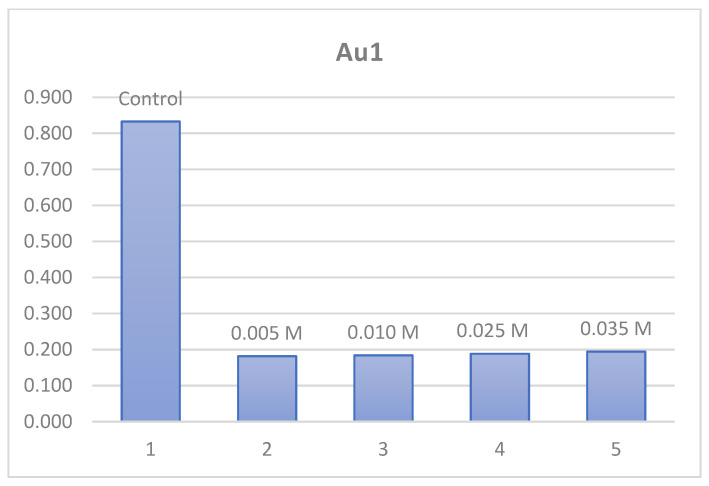
Effect of **Au1** on colon cancer cell line HT29 at different concentrations.

**Figure 7:**
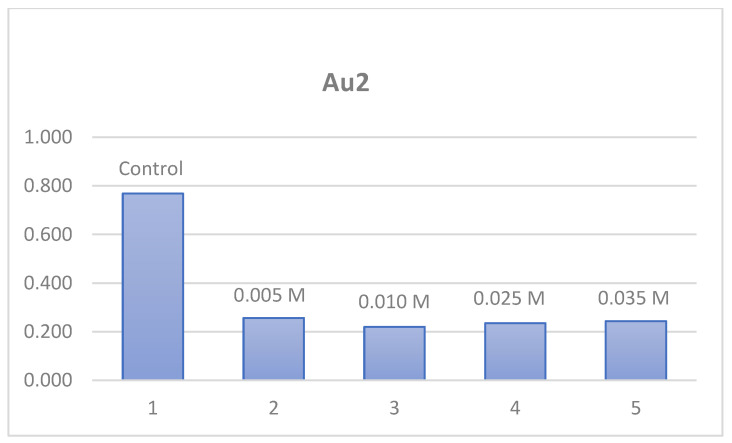
Effect of **Au2** on colon cancer cell line HT29 at different concentrations.

Different amounts of metal compound were added, and after 24 h, the MTT viability test was performed using the Cell proliferation Kit I (MTT), Roche REF number 11465007001. Cells were seeded in 96-well plates (5 × 10^4^ viable cells mL^−1^ in 100 μL), exposed to various concentrations of compounds (0.005 M, 0.010 M, 0.025 M, and 0.035 M), in medium, and incubated for 72 h. After incubation, 10 μL of MTT solution was added, and the mixture was left at 37 °C for 4 h. Then, 100 μL of solubilization buffer was added and allowed to incubate at 37 °C overnight. The absorbance was read at 620 nm by a Multiskan FC microplate photometer (Thermo Fisher Scientific IT, Milan, Italy). The percentage of cell growth was calculated by normalizing the absorbance of treated cells to corresponding control. The results indicate that increasing doses of these substances in vitro can induce morphological changes compatible with a possible cell proliferative inhibition and cell death by apoptosis (Figure 5). At the same time, no changes were evident in the viability and cytotoxicity assay (MTT) on **Au1** (Figure 6) and **Au2** (Figure 7).

Recently, a number of metal complexes have been considered as epigenetic modulators. The properties of transition metal complexes and their ability to form specific interactions with biomolecules may be responsible for the epigenetic morphological changes related with cell proliferation inhibition and apoptosis [35]. These Au complexes show a dual biological activity that is antimicrobial–antitumoral, and this aspect is appreciable for at least two reasons: (i) infecting tumoral tissues with some microorganisms makes the prognosis worse for the patient; (ii) some bacteria can turn normal tissue into cancerous tissue. Because of this, these complexes could be used to treat precancerous tissues to lower their risk of turning into cancer. However, we consider this study to be just the beginning. More tests must be performed, like tests on human non-cancerous cells and a set of in vivo tests on animal models, to confirm the activity and, most importantly, to find the best rate activity/toxicity for these compounds.

## 3. Materials and Methods

**Scheme 2:**
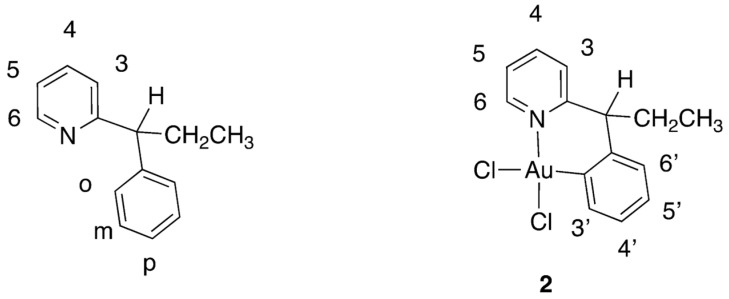
Numerical scheme.

The salt Na[AuCl_4_] was supplied by Johnson-Matthey (London, UK). The ^1^H and ^13^C NMR spectra were recorded at 400.13 and 100.61 MHz, respectively, using a Bruker (Billerica, MA, USA) Avance III 400 spectrophotometer. 2D COSY, NOESY, HSQC, and HMBC NMR spectra have been registered using standard pulse sequences. Data are reported in the following order: chemical shift (ppm, δ, with reference to TMS), multiplicity, corresponding nucleus(es), integration, and coupling constants (Hz), see Scheme 2 for numeration. Registration of the NMR spectra was carried out by Dr. Maria Orecchioni (Department of Medicine, Surgery and Pharmacy, UNISS).

Cyclic voltammetry tests were carried out in a three-electrode, single compartment cell with an AUTOLAB PGSTAT12 (Kyoto, Japan) instrument using the specific software NOVA 2.1. A Pt disk (diameter 2 mm) was the working electrode, a graphite bar was the counter electrode, and Ag/AgCl with a suitable salt bridge was the reference electrode. Before each experiment, the working electrode was polished with 1 and 0.3 μm alumina powder, then rinsed with distilled water and acetone. CH_2_Cl_2_ or CH_3_CN (anhydrous, packaged under nitrogen) was used as solvent, and 0.1 M tetraethylammonium hexafluorophosphate (TEAPF_6_) was used as supporting electrolyte. All the electrochemical experiments were performed at room temperature under argon atmosphere. All potential values are referred to the half-wave potential of the ferrocene/ferricinium ion (Fc/Fc^+^) redox couple (E_1/2_ = 0.485 and 0.430 V vs. Ag/AgCl in CH_2_Cl_2_ and CH_3_CN, respectively). Elemental analyses were performed using a LECO CHN628 Elemental Determinator (St. Joseph, MI, USA). FT-IR spectra were recorded as Nujol mulls with a JASCO (Tokyo, Japan) 4600 FT/IR spectrophotometer.

### Syntheses

**2-(1-ethylbenzil)pyridine**, **py^eb^**.

The 2-(1-phenyl-propyl)pyridine ligand was synthesized starting from 2-phenylbutyronitrile, through an acetylene co-cyclotrimerization reaction in the presence of cobalt Bonneman’s catalyst, a useful method for the build-up of pyridine rings [36].

The NMR assignments were based on H-H COSY and H-C HSQC 2D NMR experiments. ^1^H-NMR (400 MHz; CD_2_Cl_2_): δ 8.56 (ddd, *J* = 4.8, 1.8, 0.9 Hz, 1H, H^6^), 7.61 (td, *J* = 7.7, 1.9 Hz, 1H, H^4^), 7.38–7.34 (m, 2H, H^2′^), 7.33–7.28 (m, 2H, H^3′^), 7.23–7.18 (m, 2H, H^3^ + H^4′^ overlapping), 7.12 (ddd, *J* = 7.5, 4.8, 1.2 Hz, 1H, H^5^), 3.96 (t, *J* = 7.8 Hz, 1H, CH benzylic), 2.29 (m, 1H, CH_2_), 2.10 (m, *J* = 13.5, 7.4 Hz, 1H, CH_2_), 0.91 (t, 3H *J* = 7.3 Hz, CH_3_). ^13^C NMR (101 MHz; CD_2_Cl_2_): δ 163.9 (C^2^), 149.2 (C^6^), 144.2 (C^1′^), 136.2 (C^4^), 128.2 (C^3′^), 128.0 (C^2′^), 126.2 (C^4′^), 122.6 (C^3^), 121.1 (C^5^), 55.5 (CH benzylic), 28.0 (CH_2_), 12.4 (CH_3_).

**Synthesis of Au1**, **[Au(py^eb^)Cl_3_]**. An aqueous solution of Na[AuCl_4_]∙2H_2_O (103.8 mg, 0.261 mmol) in water (8 mL) was added to a solution of py^eb^ (57.1 mg, 0.289 mmol) in acetonitrile (2 mL): the resulting yellow suspension was stirred for 3 h at room temperature, then filtered under vacuum and recrystallized from CH_2_Cl_2_/Et_2_O to give complex **Au1**, [Au(py^eb^)Cl_3_], as a yellow solid (Yield 83%), m.p.: 178–180 °C. Anal. Calcd for C_14_H_15_AuCl_3_N: C 33.59; H 3.02; N 2.80%. Found: C 33.64, H 2.85, N 2.48%. The NMR assignments were based on H-H COSY and H-H NOESY 2D NMR experiments. ^1^H NMR (CDCl_3_) δ 8.68 (dd, H^6^, 1H, J_H-H_ = 6.1, 1.4 Hz); 8.04 (td, H^4^, 1H, J_H-H_ = 7.9, 1.4 Hz); 7.66 (dd, H^3^, 1H, J_H-H_ = 8.1, 1.2 Hz); 7.53 (m,H^5^, 1H); 7.46–7.38 (m, H*o*+H*m* Ph, 4H); 7.35 (m, H*p* Ph, 1H); 5.02 (dd, CH^benz^, 1H, J_H-H_ = 10.3, 4.8 Hz); 2.45–2.10 (m, H^CH2^, 2H); 1.10 (t, H^Me^, 3 H, *J* = 7.4 Hz). ^13^C NMR (CDCl_3_): 165.2 (C^2^), 149.6 (C^6^), 141.7, 138.1 (C^1′^), 129.4, 128.9, 128.2, 127.8, 125.4, 55.6 (CH benzylic), 27.9 (CH_2_), 12.4 (CH_3_). IR (Nujol, cm^−1^) 1604 s, 1595 s, 1566 s, 697 s, 366 vs. (Au-Cl). Λ_M_ (acetone, 5 × 10^−4^ M) = 1.0 ohm^−1^ cm^2^ mol^−1^ (CH_2_Cl_2_, 5 × 10^−4^ M) = 9.0 ohm^−1^ cm^2^ mol^−1^ (DMSO, 5 × 10^−4^ M) = 17.0 ohm^−1^ cm^2^ mol^−1^ (after 30′), 35.0 ohm^−1^ cm^2^ mol^−1^ (after 24h).

**Synthesis of Au2**, **[Au(py^eb^-H)Cl_2_]**. An aqueous solution of Na[AuCl_4_]∙2H_2_O (611.4 mg, 1.537 mmol) in water (24 mL) was added to a solution of py^eb^ (305.2 mg, 1.547 mmol) in acetonitrile (6 mL). The resulting yellow suspension was refluxed for 48 h, after which a white precipitate and a colorless solution were observed. The precipitate formed was filtered off, dried with Et_2_O, and recrystallized from CH_2_Cl_2_/Et_2_O to give [Au(py^eb^-H)Cl_2_], **Au2**, with an 82% yield. M.p. 240 °C. Anal. Calcd for C_14_H_14_AuCl_2_N: C 36.23; H 3.04; N 3.02%. Found: C 36.41, H 2.66, N 2.75%. The NMR assignments were based on H-H COSY, H-C HSQC and HMBC 2D NMR experiments. ^1^H NMR (CDCl_3_): δ 9.43 (d, H^6^, 1H, *J* = 5.8 Hz); 8.05 (td, 1H, *J* = 7.7 Hz, *J* = 1.4 Hz); 7.72–7.67 (m, 2H); 7.5–7.51 (m, 1H); 7.23–7.18 (m, 1H); 7.16–7.11 (m, H^6′^+H^5′^, 2H); 4.12 (t, H^benz^, 1H, *J* = 8.1 Hz); 2.73–2.55 (m, H^CH2^, 2H); 1.04 (t, H^Me^, 3 H, *J* = 7.4 Hz). ^1^H-NMR (400 MHz; CD_2_Cl_2_): δ 9.37 (m, 1H, *J* = 6.1, 1.2 Hz, H^6^), 8.09 (td, *J* = 7.7, 1.5 Hz, 1H, H^4^, 7.72 (dd, *J* = 8.0, 1.4 Hz, 1H, H^3^), 7.65 (dd, *J* = 8.0, 1.0 Hz, 1H), 7.55 (ddd, *J* = 7.6, 6.2, 1.5 Hz, 1H, H^5^), 7.23 (m, 1H, H^4^ or H^5′^), 7.18 (dd, 1H, H^6′^), 7.14 (m, 1H, H^4^ or H^5′^), 4.18 (t, *J* = 8.1 Hz, 1H, CH benzylic), 2.70–2.56 (m, 2H, CH_2_), 1.05 (t, *J* = 7.3 Hz, 3H, CH_3_). ^13^C NMR (CD_2_Cl_2_): 158.44 (C^2^), 152.21 (C^6^), 142.19 (C^4^), 139.88 (C^2′^-Au), 134.35 (C^1′^), 133.46 (C^3′^), 129.18 (C^6′^), 128.07 (C^4^ or C^5′^), 127.71 (C^4^ or C^5′^), 126.96 (C^3^), 124.64 (C^5^), 60.91 (CH benzylic), 32.99 (CH_2_), 12.39 (CH_3_). IR (Nujol, cm^−1^) 1604 s, 1578 s, 1564 s, 351 vs. and 302 vs. (Au-Cl). Λ_M_ (CH_2_Cl_2_, 5 × 10^−4^ M) = 0.2 ohm^−1^ cm^2^ mol^−1^ (DMSO, 5 × 10^−4^ M) = 4.2 ohm^−1^ cm^2^ mol^−1^_._

## 4. Conclusions

In this paper, we have reported the synthesis and characterization of two structurally different gold(III) complexes with nitrogen ligands, i.e., a simple N-coordinated adduct (**Au1**) and a firmly coordinated, stable N^C cyclometalated complex, **Au2**. In addition to the characterization of these two complexes, we have also investigated some aspects of their biological behavior, testing some antimicrobial and, preliminarily, also antitumor properties.

Over the past decade, indeed, there has been an increased interest in identifying alternative ways to fight bacterial infections and to address the antimicrobial resistance (AMR) health crisis. Metal ions have a long history of antimicrobial activity and have received increasing attention in recent years. Although they have not yet been brought into the clinical setting, metalloantibiotics are a large and under-explored group of compounds that could lead to a much-needed new class of antibiotics. There are far fewer reports on the antimicrobial activity of gold complexes, despite the fact that metal-based compounds represent a very promising chemical scaffold in this field, as they can act against non-classical targets and multiple bacterial sites simultaneously. In this context, some authors have recently reported on antimicrobial activity against Gram-positive species such as Staphylococcus. Following these previous studies, we report here on a series of coordination compounds with gold of the general formula [Au(py^eb^)Cl_3_] and [Au(py^eb^-H)Cl_2_] (**Au1** and **Au2**, respectively) as promising antibacterial and antibiofilm compounds against Gram-positive and, more importantly, Gram-negative bacteria such as *K*. *pneumoniae* and *E*. *coli*.

Despite the different structures of the monodentate adduct **Au1** and the cyclometalated complex **Au2**, these results demonstrated that the compounds have similar antibacterial activity: they in fact inhibited the same strains. The results show an interesting antibiofilm activity of the Au1 complex against the Gram-negative pathogen *K. pneumoniae*, a cause of nosocomial infections, while the Au2 complex showed higher antibiofilm activity against the anaerobic pathogen *S. pyogenes*, responsible for pharyngitis.

The two complexes were deeply investigated by means of NMR and cyclic voltammetry in solutions, showing very different levels of robustness. The cyclometalated complex **Au2** appears to be very stable in solution, whereas the adduct **Au1** seems more prone to reduction and overall transformations.

The results of tests on antimicrobial activity suggest that the analyzed compounds have the ability to prevent the growth and formation of biofilms of pathogenic microorganisms and that **Au1** and **Au2** could be raw materials for obtaining new antimicrobials.

The next step will be to understand the mode of action involved in their antimicrobial and antibiofilm activity against different microbial species.

The different levels of chemical and electrochemical robustness found for the two complexes, whose solutions showed similar antimicrobial activity, may be exploited in the future for different purposes, taking advantage of their chemical diversity but similar antimicrobial effects.

The preliminary evaluation of the in vitro activity of **Au1** and **Au2** against the HT29 colon cancer cell line also yielded very promising results.

## Figures and Tables

**Table 1:** Antimicrobial profile for **Au1** [Au(py^eb^)Cl_3_] and **Au2** [Au(py^eb^-H)Cl_2_] 0.01 M with a set of Gram-positive and Gram-negative bacteria and yeast.

Strain	[Au(py^eb^)Cl_3_] (Au1)	[Au(py^eb^-H)Cl_2_] (Au2)	Control
Inhibition Diameter Ø mm			
Gram-negative			
*E*. *coli*	17 ± 0.0	16 ± 0.0	Amp. 19.6 ± 0.7
*K*. *pneumoniae*	16 ± 0.0	15 ± 0.0	Amp. 9.5 ± 0.8
Gram-positive			
*S*. *aureus*	14 ± 0.0	20 ± 0.0	Ox. 22.6 ± 1.5
*S*. *pyogenes*	25 ± 0.0	22 ± 0.0	Amp. 23.8 ± 0.9
Yeast			
*C*. *albicans*	0.0 ± 0.0	0.0 ± 0.0	KTC 20 ± 1

Legend: Amp. = ampicillin; KTC = Ketoconazole; Ox. = oxacillin.

**Table 2:** Minimum inhibitory concentration (MIC), minimum bactericide concentration (MBC) and antibiofilm assay (MBIC) values observed with a set of Gram-positive and Gram-negative bacteria.

Strain	Compound	MIC	MBC	MBIC
*E*. *coli*	[Au(py^eb^)Cl_3_] [Au(py^eb^-H)Cl_2_]	6.2 × 10^−4^ 6.2 × 10^−4^	6.2 × 10^−4^ 6.2 × 10^−4^	6.2 × 10^−4^ 6.2 × 10^−4^
*K*. *pneumoniae*	[Au(py^eb^)Cl_3_] [Au(py^eb^-H)Cl_2_]	6.2 × 10^−4^ 1.5 × 10^−4^	6.2×10^−4^ 1.5 × 10^−4^	1.25 × 10^−3^ 2.5 × 10^−3^
*S*. *aureus*	[Au(py^eb^)Cl_3_] [Au(py^eb^-H)Cl_2_]	6.2 × 10^−4^ 1.5 × 10^−4^	6.2 × 10^−4^ 1.5 × 10^−4^	2.5 × 10^−3^ 1.5 × 10^−4^
*S*. *pyogenes*	[Au(py^eb^)Cl_3_] [Au(py^eb^-H)Cl_2_]	1.25 × 10^−3^ 6.2 × 10^−4^	1.25 × 10^−3^ 6.2 × 10^−4^	6.2 × 10^−4^ 6.2 × 10^−4^

## Data Availability

The data that support the findings of this study are available from the corresponding authors upon reasonable request.

## Supplementary Materials

The following supporting information can be downloaded at: https://www.mdpi.com/article/10.3390/molecules30071611/s1, Figures S1–S17: NMR spectra of complexes **Au1** and **Au2** and Cyclic voltammetric curves of **Au1**.

## Abbreviations

The following abbreviations are used in this manuscript:

MDPI	Multidisciplinary Digital Publishing Institute
DOAJ	Directory of open access journals
TLA	Three-letter acronym
LD	Linear dichroism
