# Supplementary material for: Gold(III) Complexes with 2-(1-Ethylbenzyl)pyridine as Promising Antimicrobial and Antitumor Agents
Source: Molecules. 2025 Apr 4;30(7):1611. doi: 10.3390/molecules30071611 (PMC11990560; doi:10.3390/molecules30071611)
Supplement: Supplementary file 1 [file molecules-30-01611-s001.zip › molecules-3545037-supplementary.pdf]

# Gold(III) complexes with 2-(1-ethylbenzyl)pyridine as promising antimicrobial and antitumor agents

**Antonio Zucca [1,2] *, Bruna Canu[1], Maria I. Pilo,[1] Sergio Stoccoro[1,2], Giacomo Senzacqua[1,2], Sara Fais[3], Giuseppina Pichiri[4], and Alessandra Scano [3,*]**

[1] Department of Chemical, Physical, Mathematical and Natural Sciences, University of Sassari, Via Vienna 2, 07100 Sassari, Italy
[2] Consorzio Interuniversitario Reattività Chimica e Catalisi (CIRCC) Bari (Italy
[3] Department of Surgical Sciences, University of Cagliari, 09124 Cagliari, Italy
[4] Department of Medical Sciences and Public Health, University of Cagliari, 09124, Cagliari, Italy.

## Supporting Material

| | |
|---|---|
| Figures S1-S5 | NMR spectra of complex **Au1** |
| Figures S6-S16 | NMR spectra of complex **Au2** |
| Figure S17 | Cyclic voltammetric curves of **Au1** |

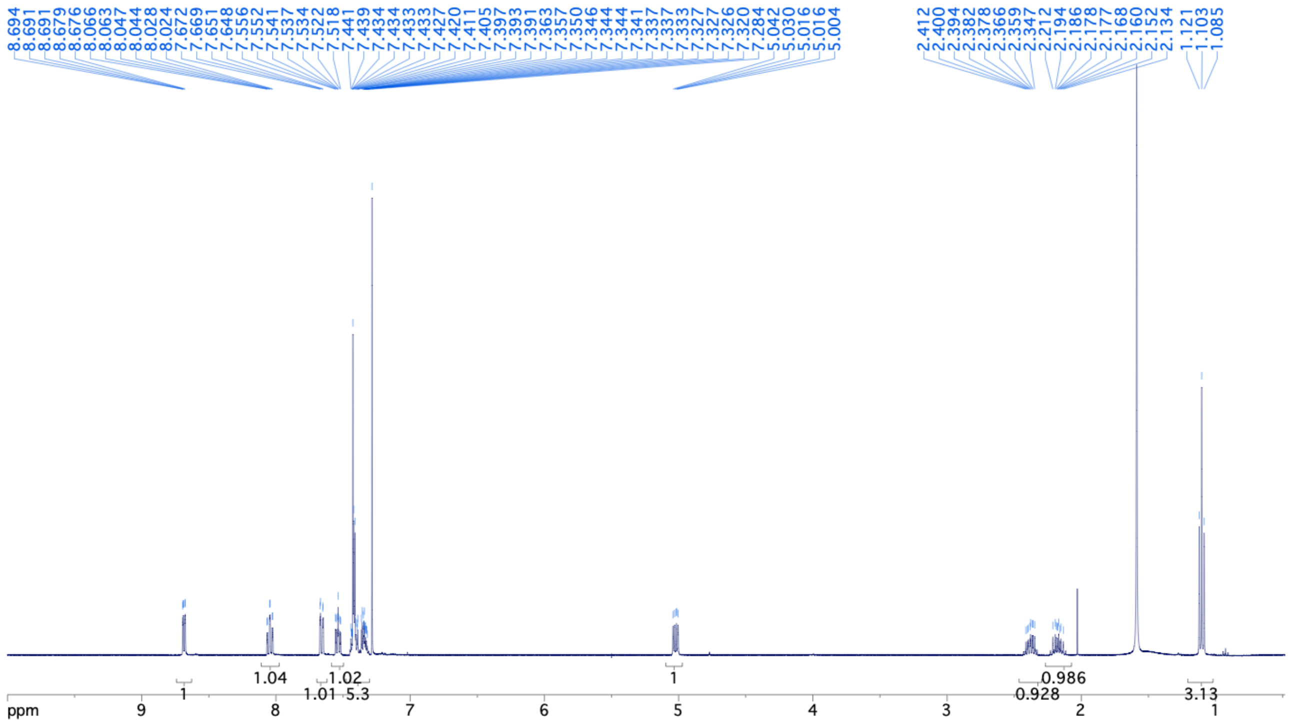

Figure S1. $^1$H NMR spectrum of complex **Au1**($CDCl_3$)

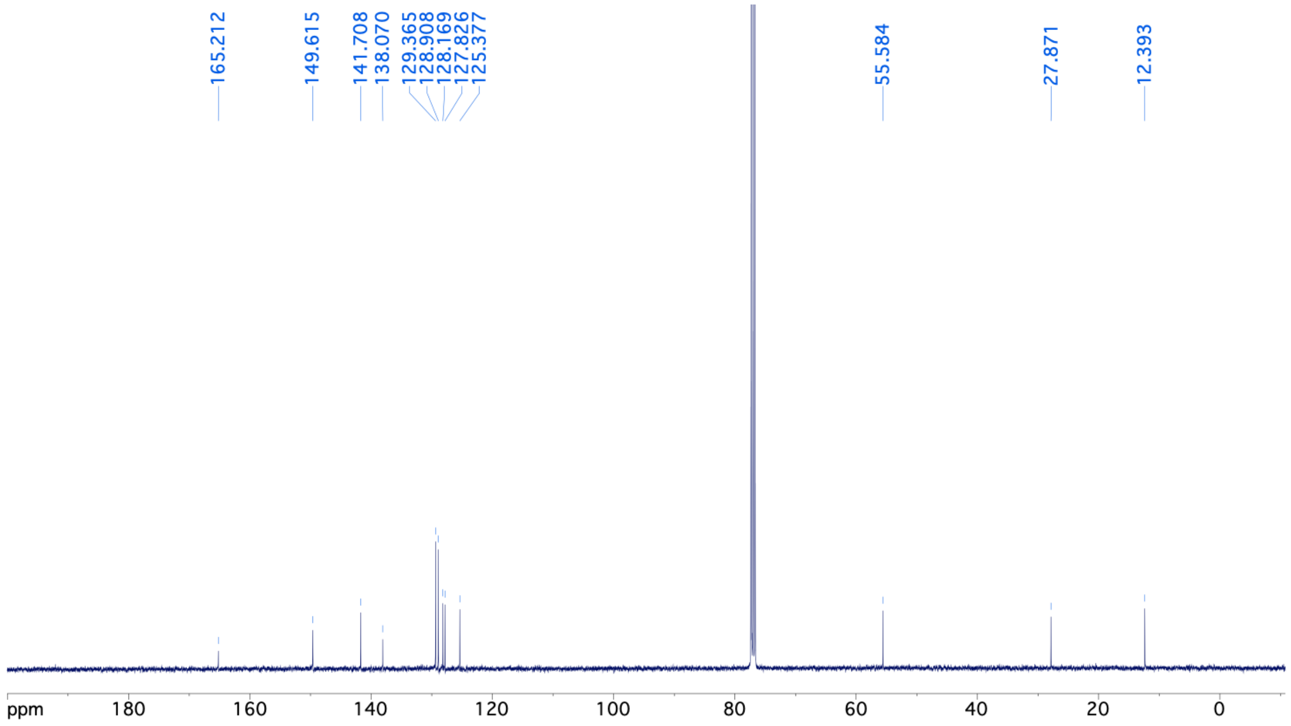


Figure S2. $^{13}C$ NMR spectrum of complex **Au1**($CDCl_3$)

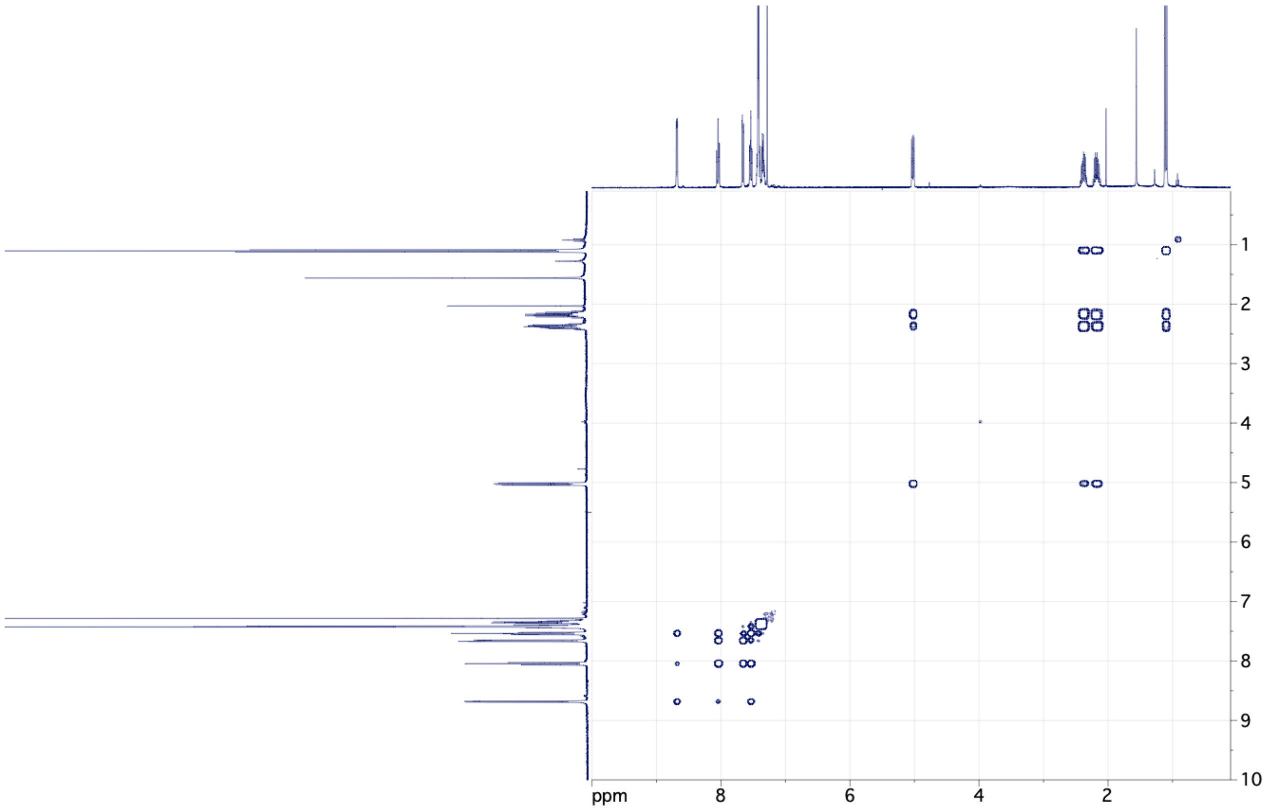


Figure S3. 2D $^1$H COSY NMR spectrum of complex **Au1** ($CDCl_3$)

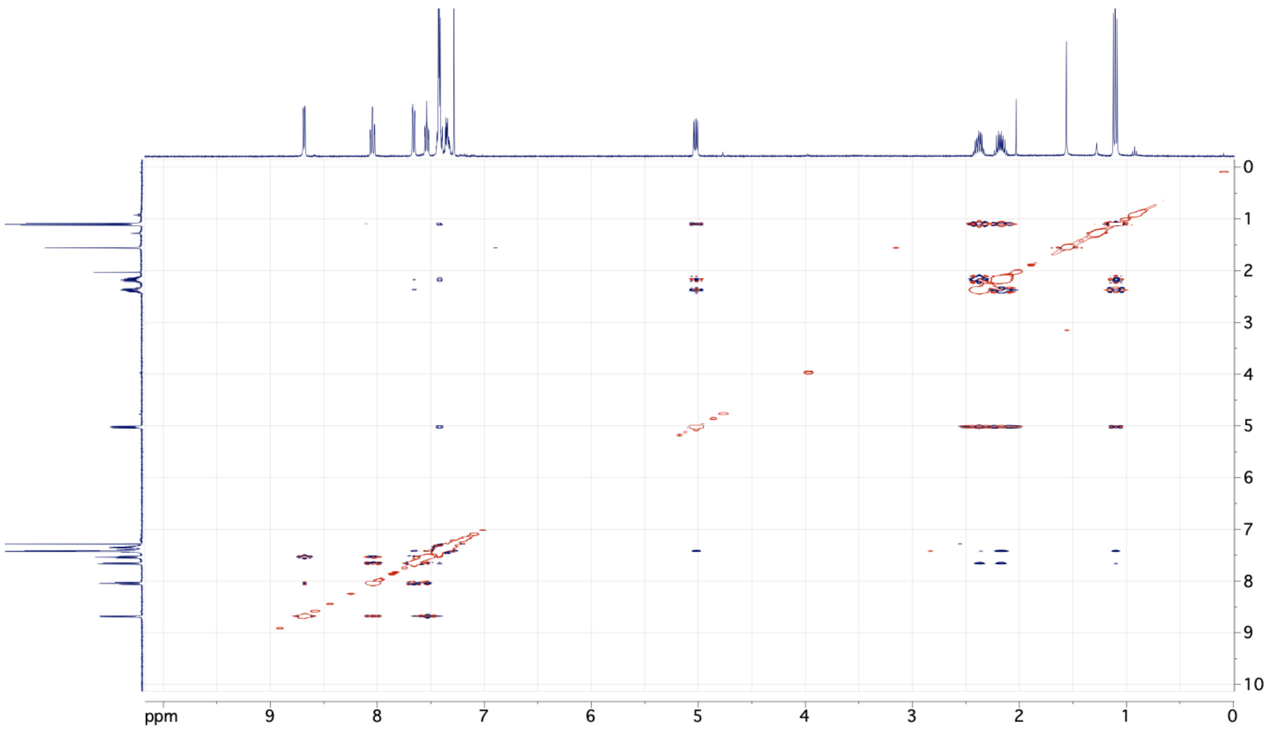


Figure S4. 2D $^{1}H$ NOESY NMR spectrum of complex **Au1** ($CDCl_3$)

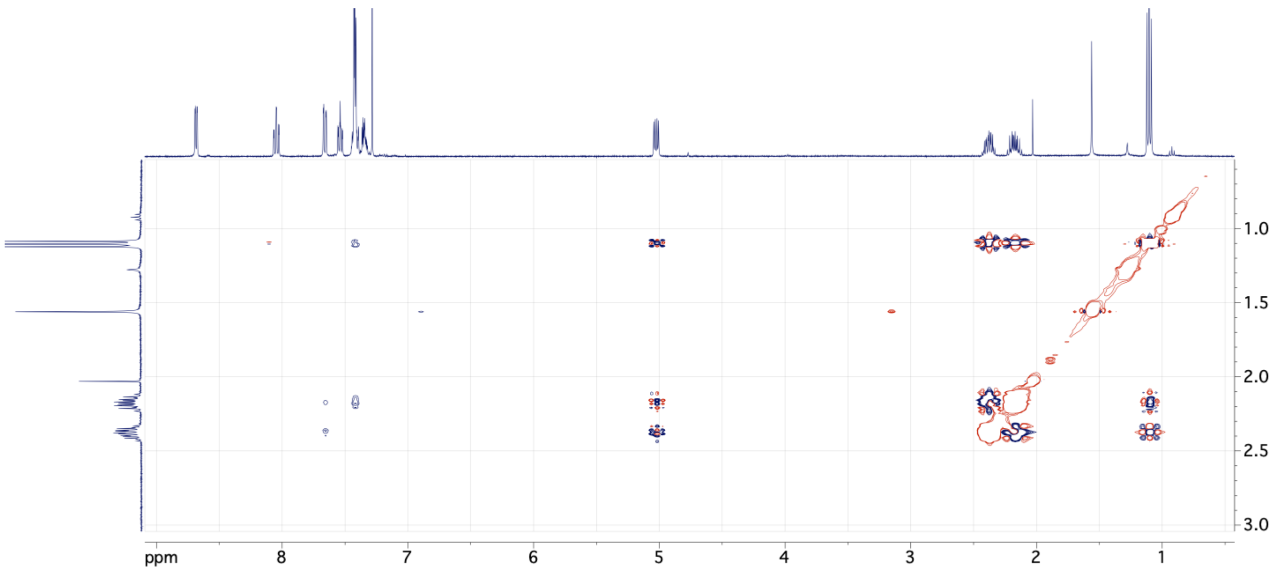


Figure S5. Section of the 2D $^{1}H$ NOESY NMR spectrum of complex **Au1** ($CDCl_3$)

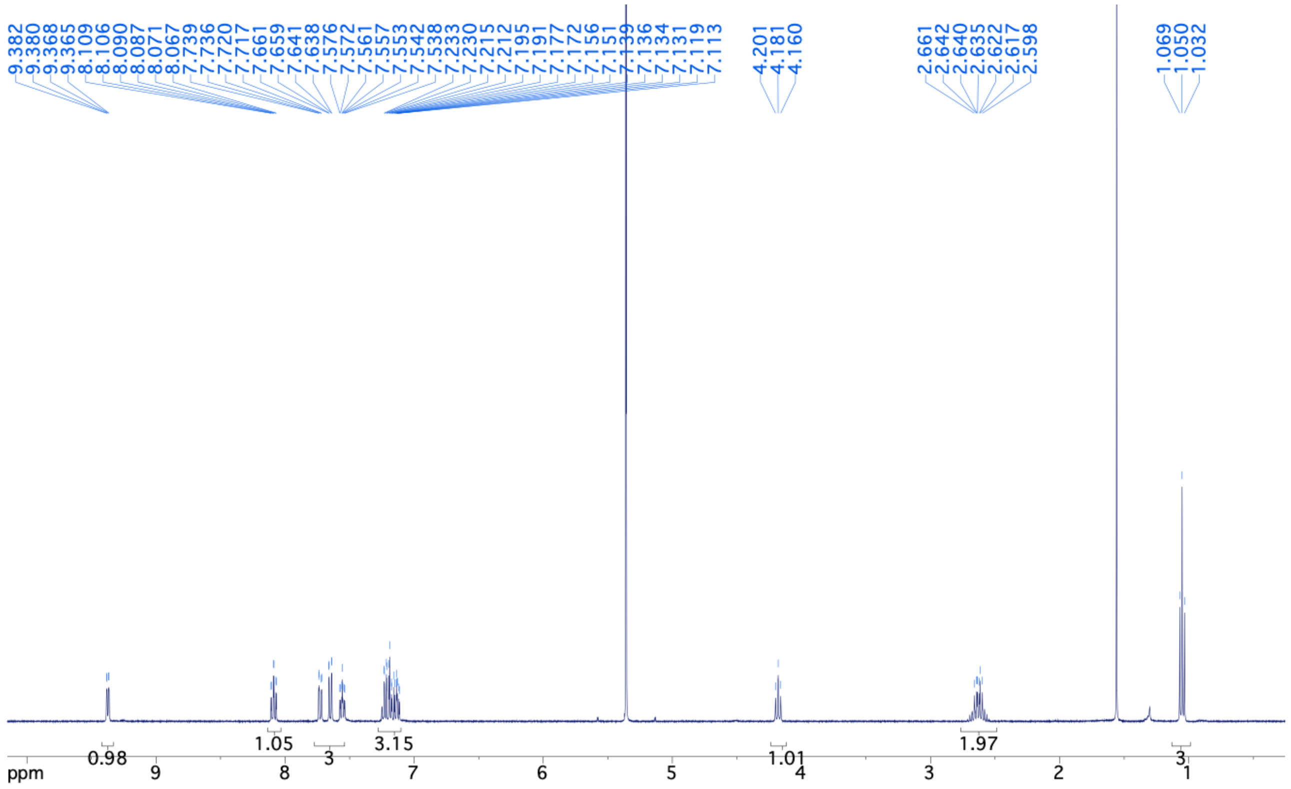


Figure S6. $^{1}$H NMR spectrum of complex **Au2** ($CD_2Cl_2$)

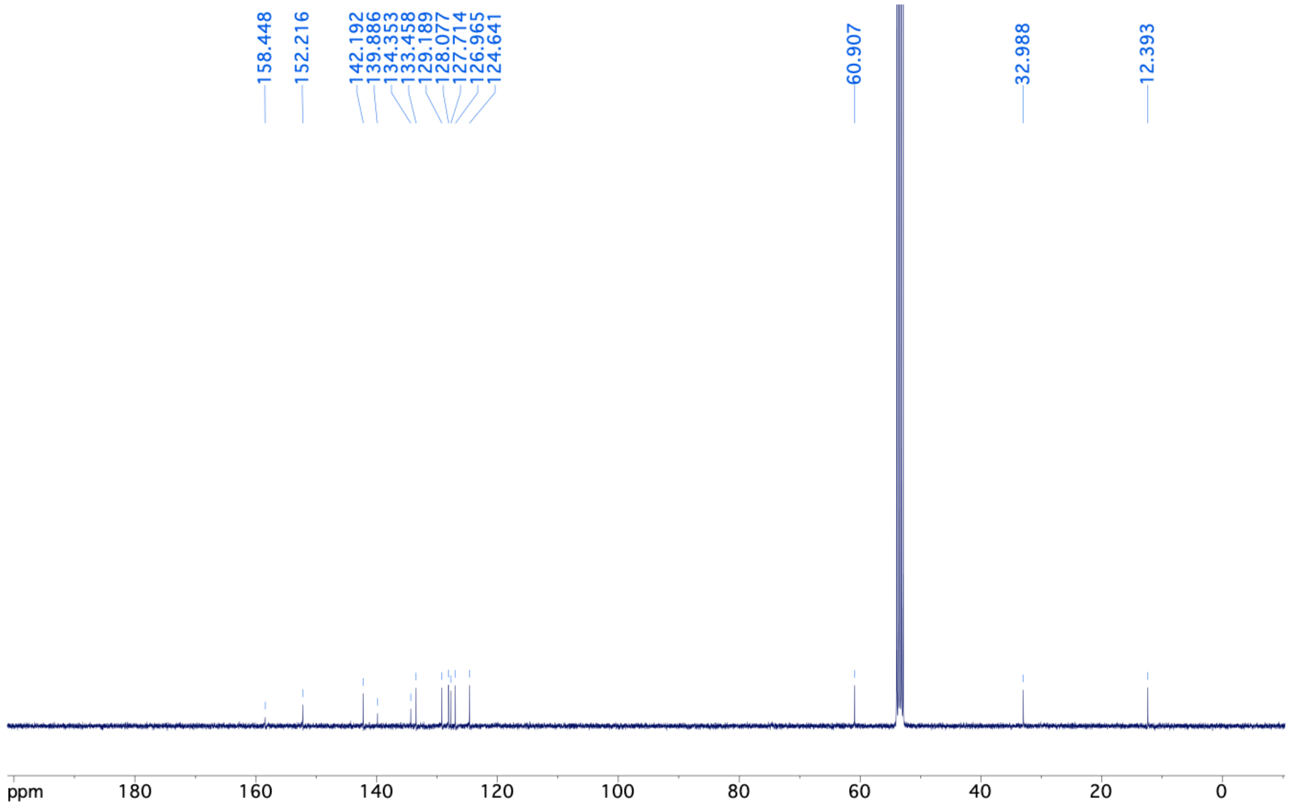


Figure S7. $^{13}$C NMR spectrum of complex **Au2** ($CD_2Cl_2$)

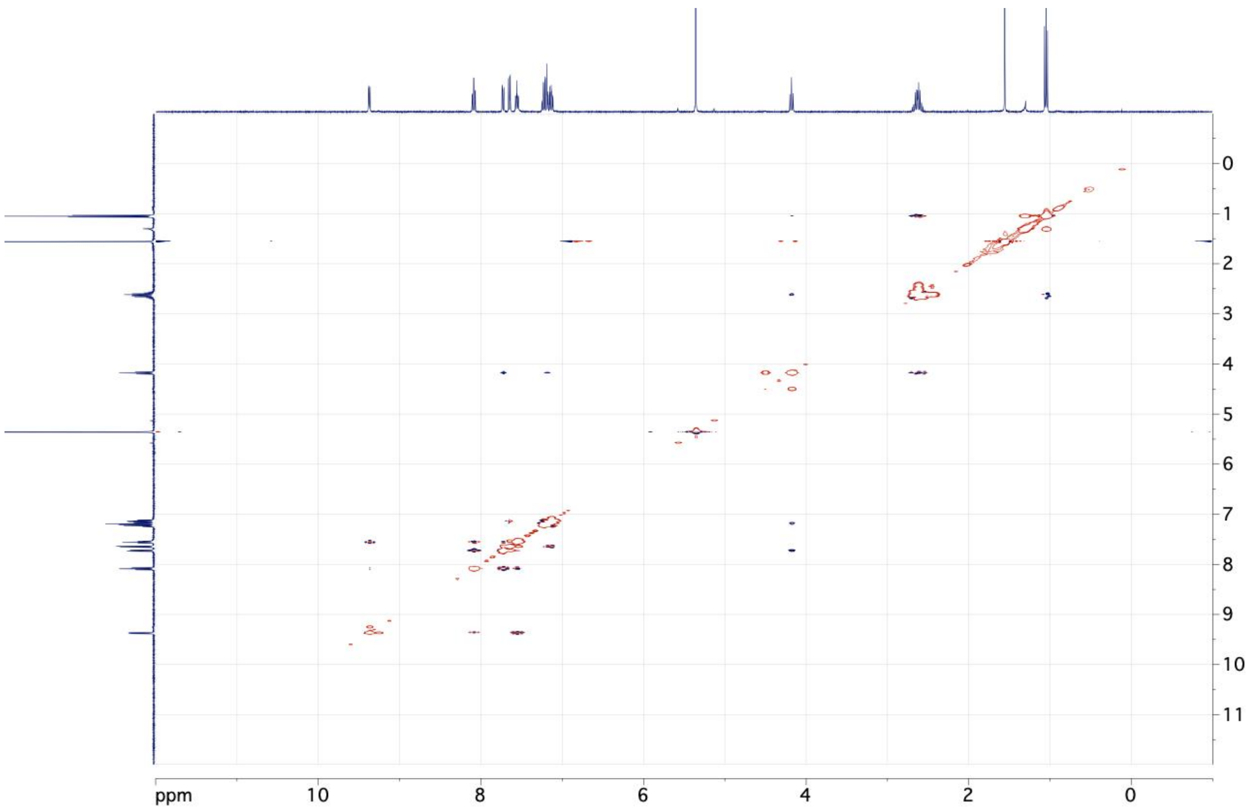


Figure S8. 2D $^{1}$H NOESY/EXSY NMR spectrum of complex **Au2** ($CD_2Cl_2$)

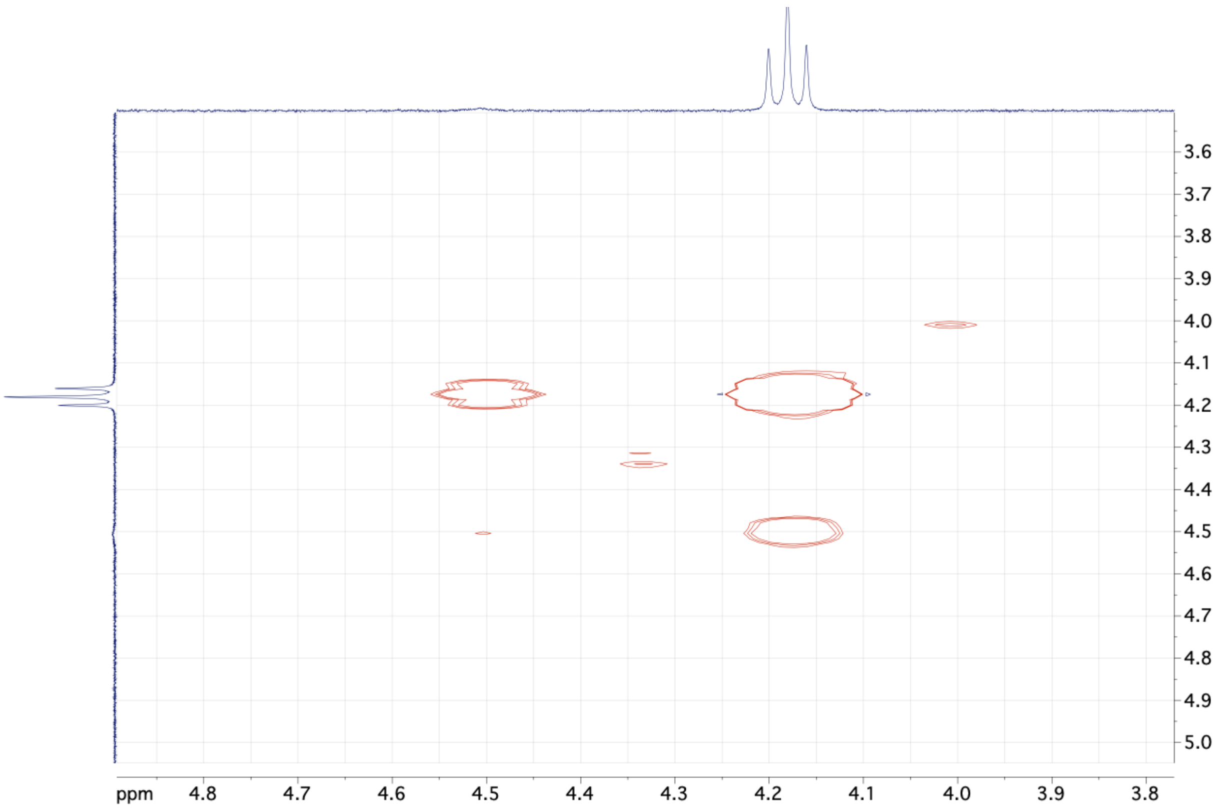


Figure S9. Section of the 2D $^{1}$H NOESY/EXSY NMR spectrum of complex **Au2** ($CD_2Cl_2$)

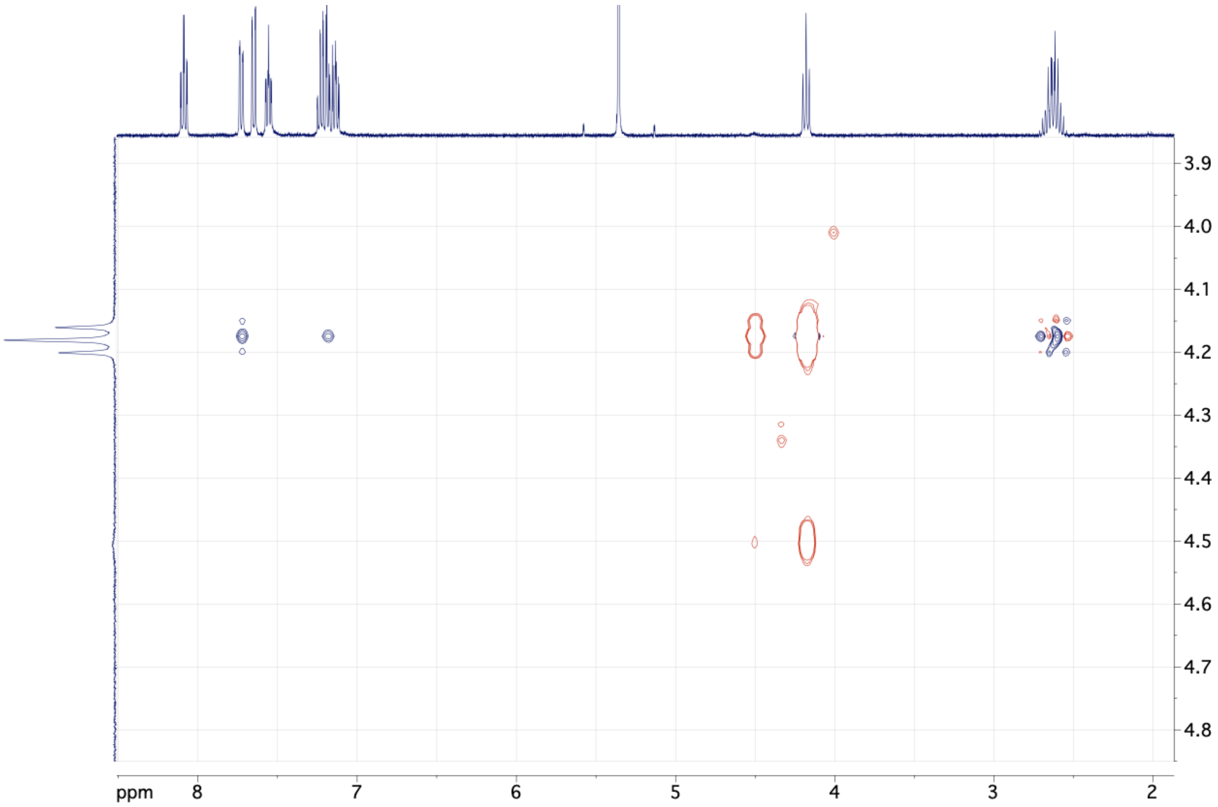


Figure S10. Section of the 2D $^{1}H$ NOESY/EXSY NMR spectrum of complex **Au2** ($CD_2Cl_2$)

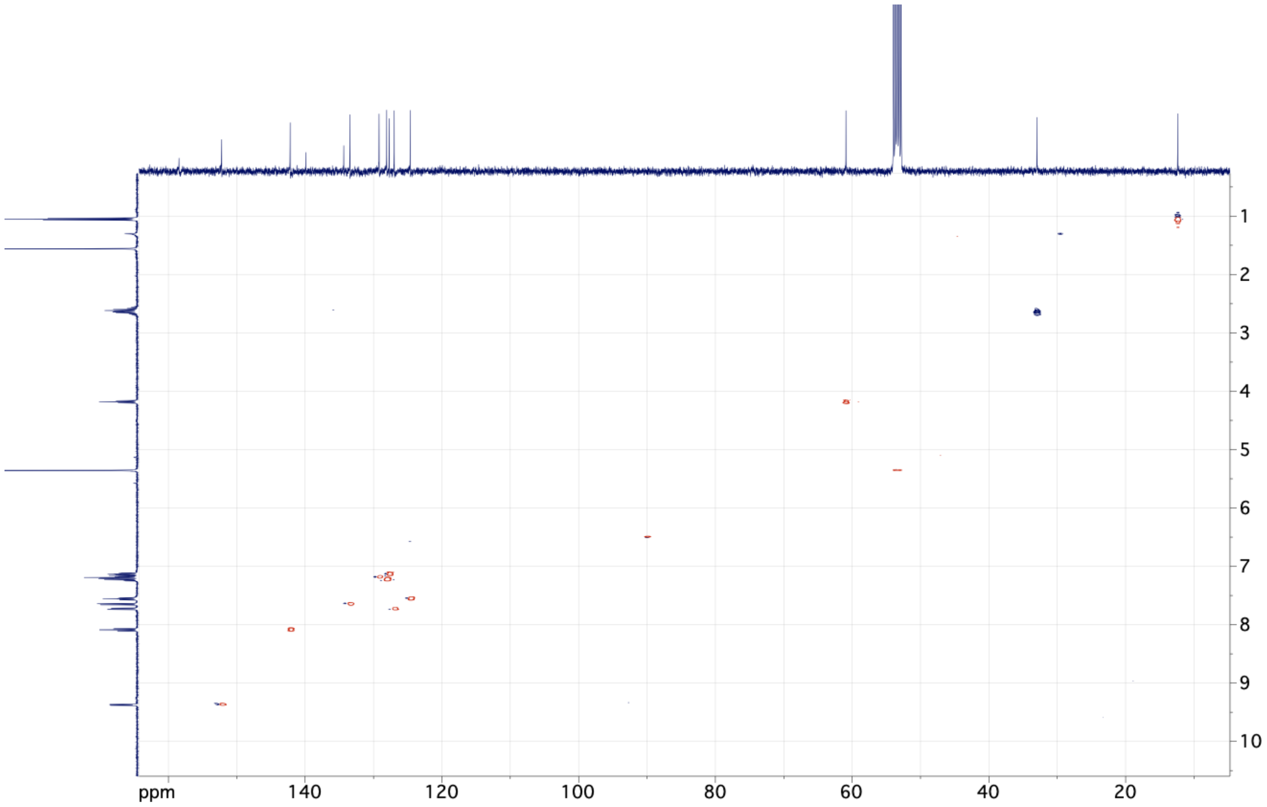


Figure S11. $^{1}H$-$^{13}C$ HSQC NMR spectrum of **Au2** ($CD_2Cl_2$)

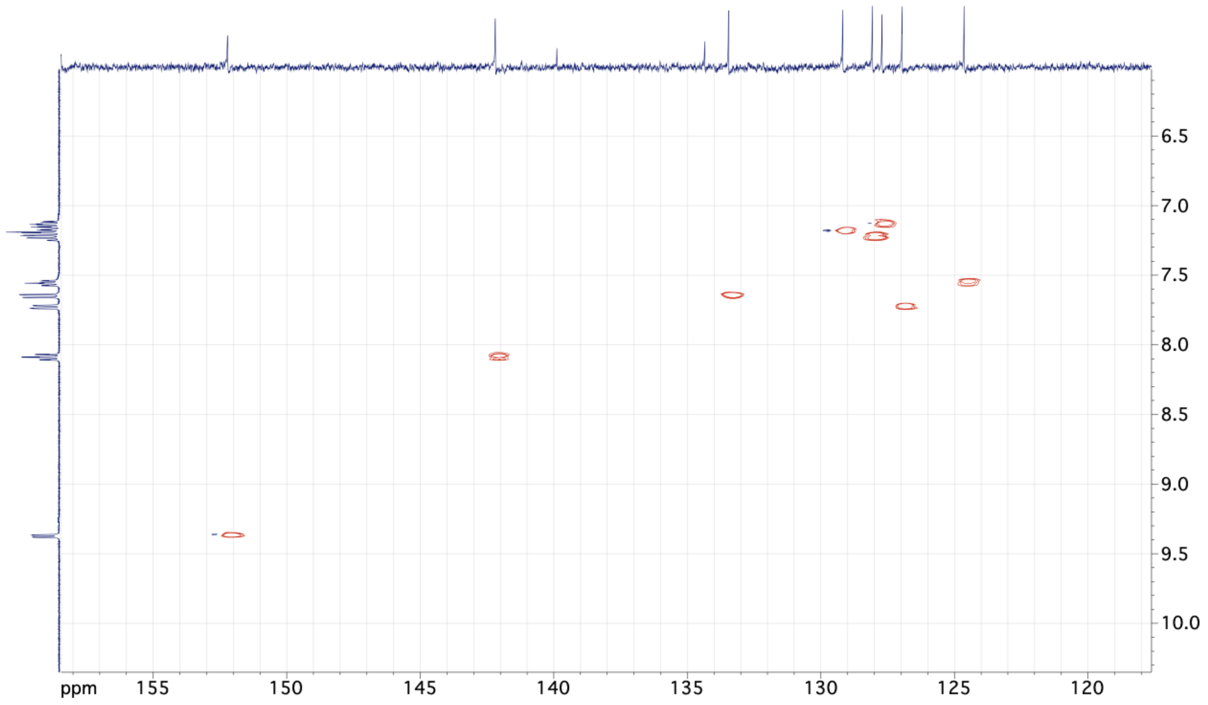


Figure S12. Section of the $^{1}H$-$^{13}C$ HSQC NMR spectrum of **Au2** ($CD_2Cl_2$)

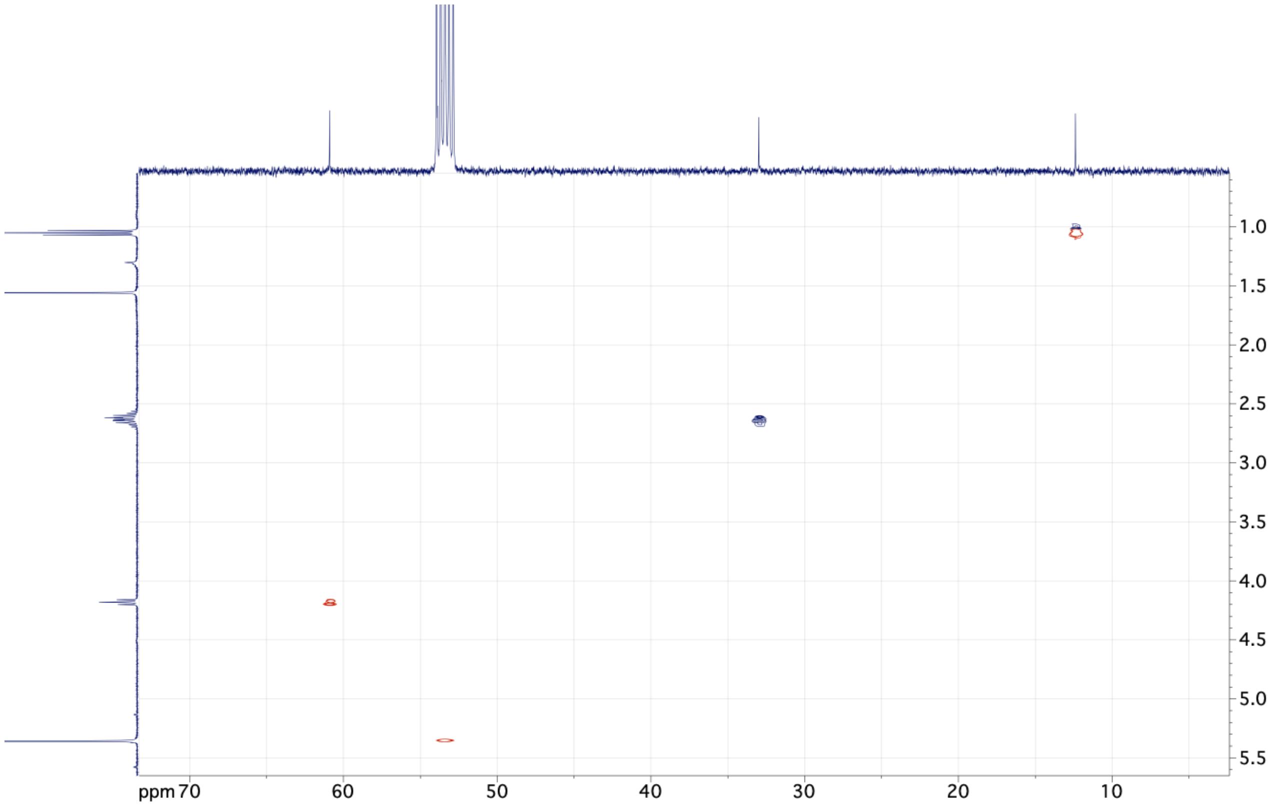


Figure S13. Section of the $^{1}H$-$^{13}C$ HMBC NMR spectrum ($CD_2Cl_2$)of **Au2**

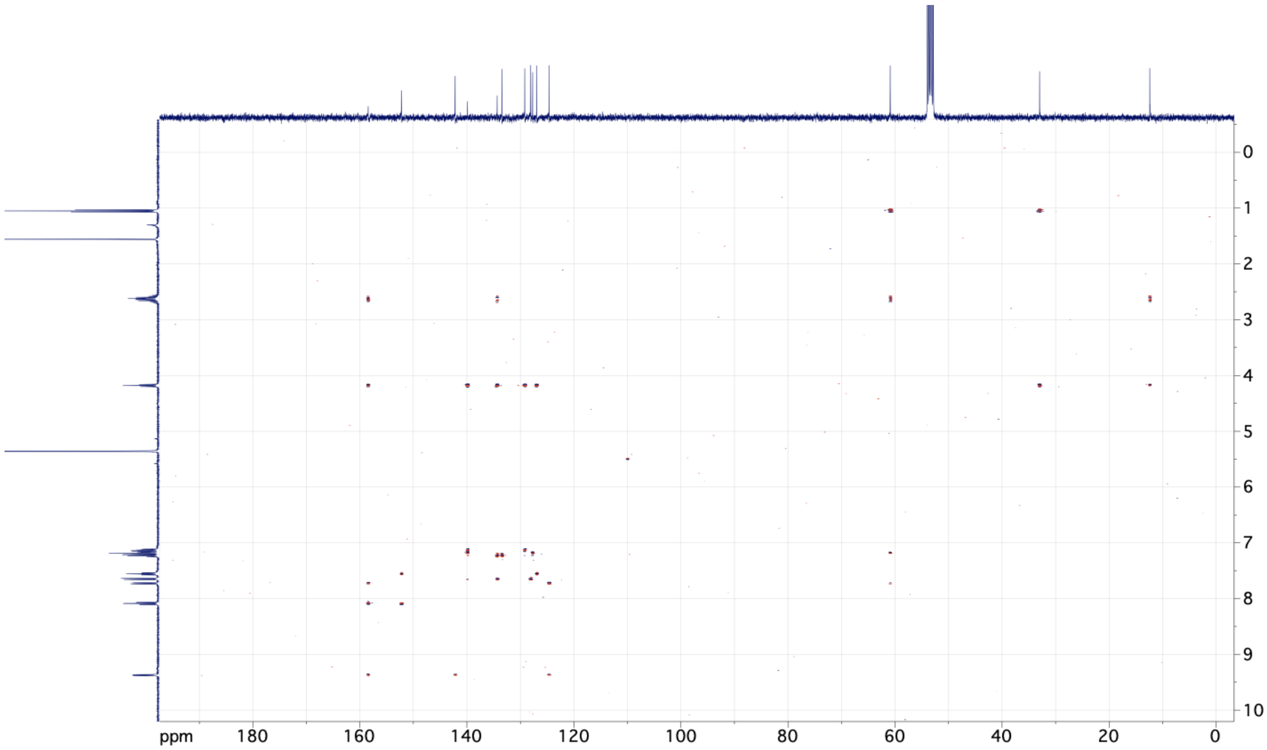


Figure S14. $^{1}H$-$^{13}C$ HMBC NMR spectrum of **Au2** ($CD_2Cl_2$)

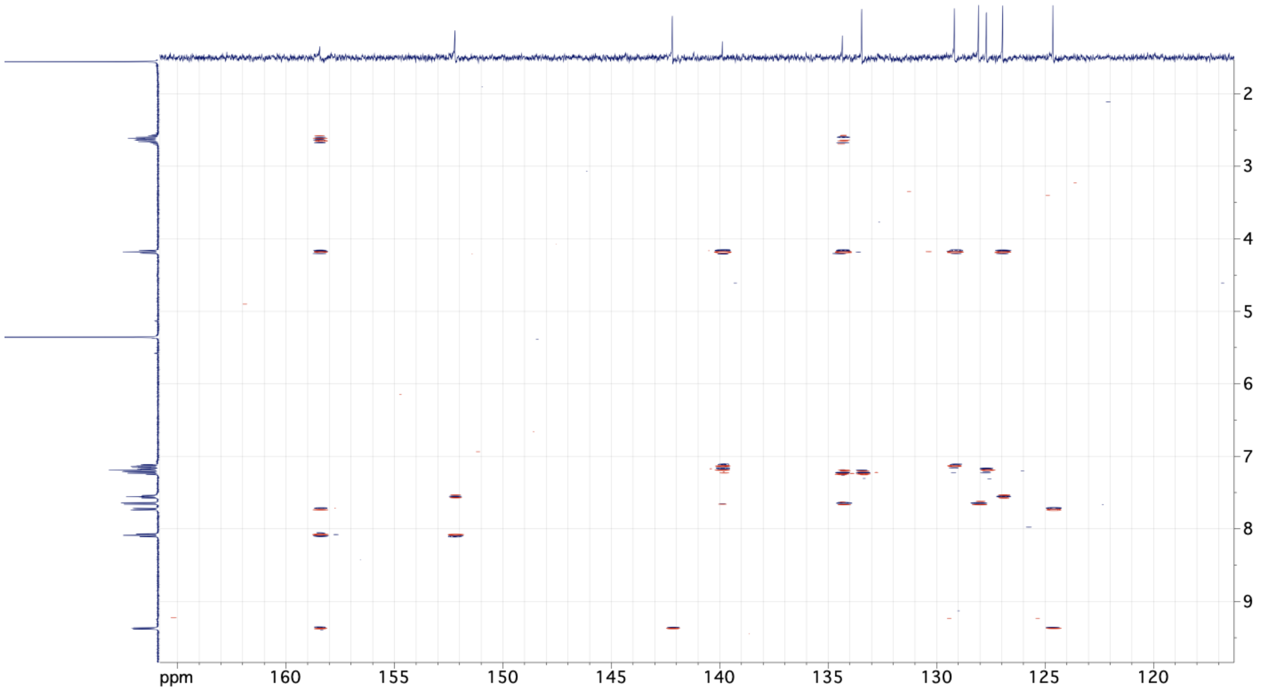


Figure S15. Section of the $^{1}H$-$^{13}C$ HMBC NMR spectrum of **Au2** ($CD_2Cl_2$)

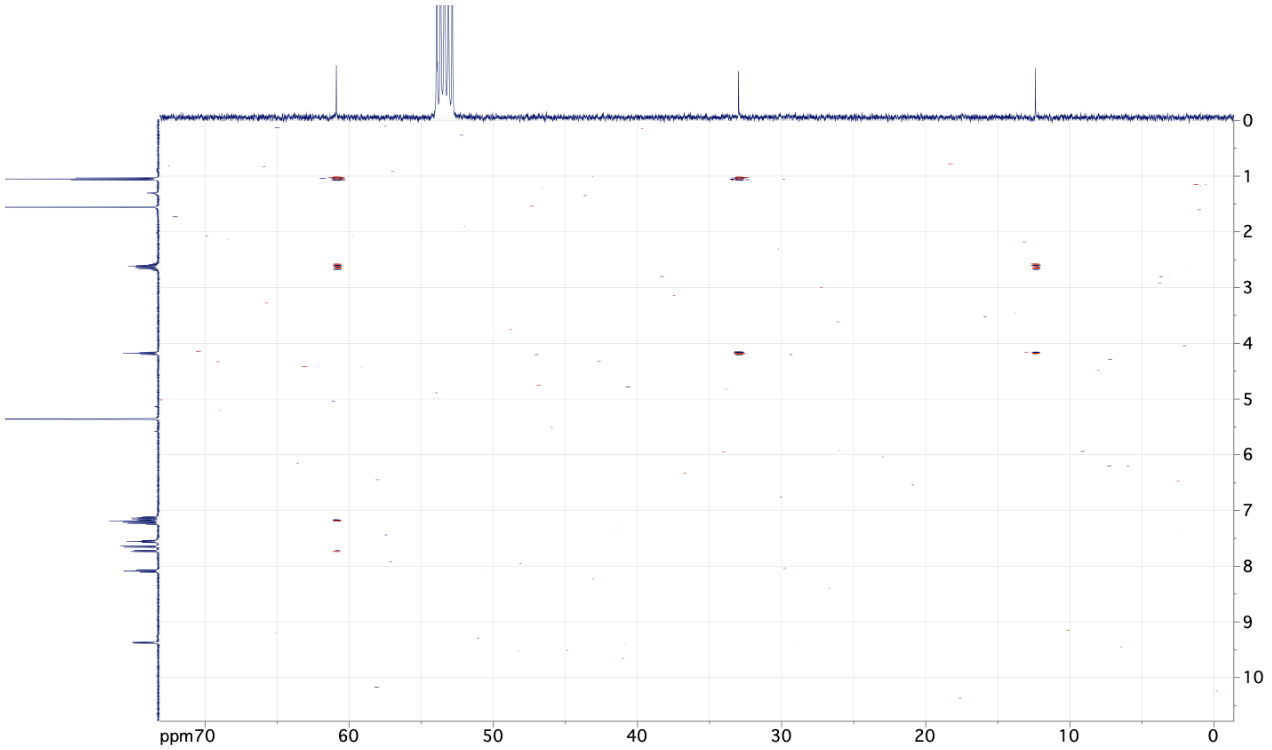


Figure S16. Section of the $^{1}H$-$^{13}C$ HMBC NMR spectrum of **Au2** ($CD_2Cl_2$)

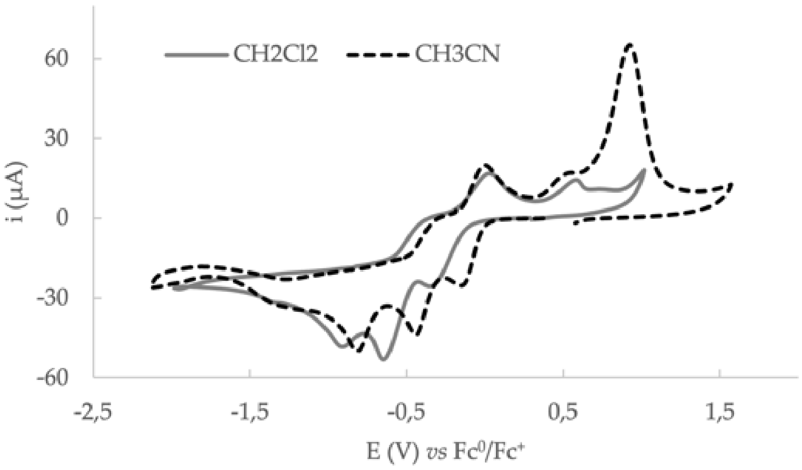


**Figure S17.** Cyclic voltammetric curves of **Au1** at 0.20 V $s^{-1}$ in methylene chloride (continuous curve) and in acetonitrile (dotted curve) as the solvent.
